# General Spin-Restricted Open-Shell Configuration Interaction Approach: Application to Metal K-Edge X-ray Absorption Spectra of Ferro- and Antiferromagnetically Coupled Dimers

**DOI:** 10.1021/acs.jpca.4c05228

**Published:** 2024-12-16

**Authors:** Tiago Leyser da Costa Gouveia, Dimitrios Maganas, Frank Neese

**Affiliations:** Max-Planck-Institut für Kohlenforschung, Kaiser-Wilheim-Platz 1, 45470 Mülheim an der Ruhr, Germany

## Abstract

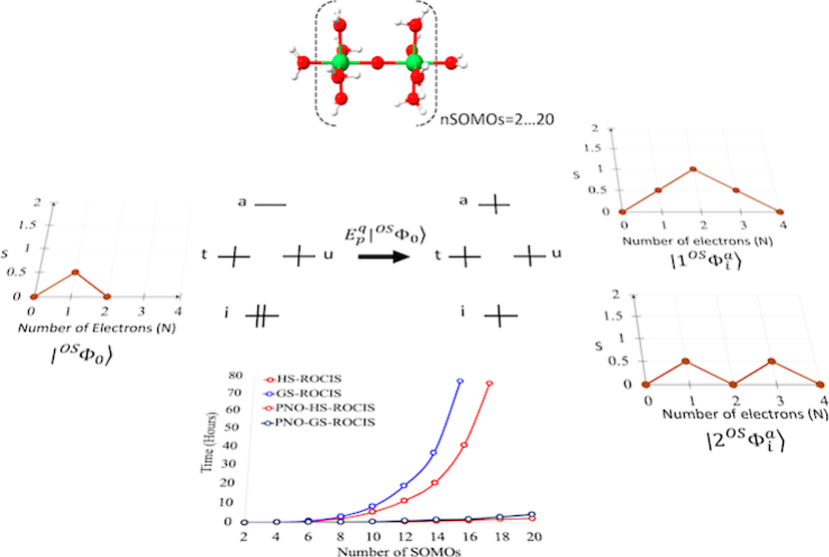

In this work, we present a generalized implementation of the previously developed restricted open-shell configuration interaction singles (ROCIS) family of methods. The new method allows us to treat high-spin (HS) ferro- as well as antiferromagnetically (AF) coupled systems while retaining the total spin as a good quantum number. To achieve this important and nontrivial goal, we employ the machinery of the iterative configuration expansion (ICE) method, which is able to tackle general configuration interaction (CI) problems on the basis of spin-adapted configuration state functions (CSFs). While ICE is designed to work in restricted orbital spaces, the new general-spin ROCIS (GS-ROCIS) method is designed to be applicable to larger molecules by employing a prototyping strategy. This new method can be applied to closed-shell, high-spin open-shell, as well as antiferromagnetic reference CSFs. In addition, GS-ROCIS can be combined with the pair natural orbital (PNO) machinery in the form of the PNO-GS-ROCIS method. With this extension, one can drastically reduce the required virtual space in the vicinity of the involved core orbitals, leading to computational savings of several orders of magnitude with negligible (<1%) loss in accuracy. To demonstrate the use of the new methodology, the metal K pre-edge X-ray absorption excitation problem of an antiferromagnetically coupled copper model dimer was investigated. By first analyzing a model copper dimer, it is shown that even for the minimum core excitation problem that involves the two antiferromagnetically coupled singly occupied orbitals and one virtual orbital, the resulting GS-ROCIS and broken-symmetry configuration interaction singles (BS-CIS) spectra may differ in terms of the number, energy position, and relative intensity of the computed bands. Furthermore, the methodology was validated to perform equally well in computing the K-edge spectra of antiferromagnetic nickel oxide dimers and mixed-valence cobalt oxide trimers. Collectively, the present development represents an important methodological advance in the application of theoretical X-ray spectroscopy.

## Introduction

Over the last decades, X-ray absorption spectroscopy (XAS) has been established as a powerful analytical tool that is in large-scale use in all branches of chemistry that deal with transition metals and other heavier elements.^1−15^ The key advantage of XAS and related techniques is that they are element-specific and offer a wealth of information about the local geometric and electronic structures around the absorber atom(s). Owing to this high degree of selectivity that is offered by the element-specific nature of the X-ray techniques, XAS has proven to be instrumental in probing the local coordination environment, as well as spin and oxidation states of centers in extended polymetallic networks.^10,16−21^ In particular, XAS spectroscopy has helped to identify the spin and oxidation states of multimetallic catalytic active-site networks as those met in the cuboid Mn active site of the oxygen-evolving complex,^10,16,17,22^ the FeMoco center in nitrogenase,^9,11,23^ and metal oxide-based solid catalysts.^24−31^

The quantitative interpretation of such spectra from first principles is a challenging task that is perhaps most adequately approached by employing wave function-based methodologies. In particular, wave function techniques provide an explicit treatment of ligand field splittings, metal–ligand covalency effects, and, most importantly, multiplet effects. An exact treatment of the involved multiplet effects requires the use of properly spin- and space-symmetry-adapted configuration state functions (CSFs), which is currently only possible within wave function-based theories. Importantly, wave function-based techniques allow for a detailed and physically correct treatment of spin–orbit coupling phenomena through an explicit representation of the entire manifold of the magnetic sublevels (*M*_S_ components) arising from all the involved multiplets. A potential drawback of wave function-based methods in the field of X-ray spectroscopy is the fact that quantitatively accurate results require taking account of electron correlation, which quickly leads to bottlenecks with respect to system size and computational complexity. Hence, it is highly desirable to develop approximate methods that can be applied to larger systems and, through embedding techniques, also to extended systems.

Numerous studies exist, demonstrating that wave function-based protocols employing the complete or the restricted active space configuration interaction methods (CASSCF/RASSCF^32−35^) in conjunction with N-electron valence second-order perturbation theory (NEVPT2^36,37^) or restricted active space perturbation theory (RASPT2^38^), namely, CASCI/NEVPT2 and RASSCF/RASPT2, have shown excellent performance in computing XAS,^39^ X-ray magnetic circular dichroism (XMCD),^40^ and resonance inelastic X-ray scattering (RIXS) spectra of medium-size molecules.^41^ Furthermore, several studies exist employing multireference configuration interaction (MRCI) to calculate XAS, XES, and RIXS^42^ spectra, while recently, multireference equation of motion coupled cluster (MREOM-CC) methods have been employed to compute the challenging metal L-edge XAS spectra of small iron complexes.^43^ In addition, variants of the equation of motion coupled cluster (EOM-CC),^44−46^ as well as linear response CASSCF,^47^ have been employed to compute XAS spectra of small molecules. However, it should be emphasized that the number of final states that one needs to consider in X-ray absorption techniques can easily reach hundreds to thousands. The requirements to represent the entire final states drastically limit the applicability of most methods as the system size of the studied system increases.

An alternative approach, which is also based on wave function theory, is provided by the restricted open-shell configuration interaction singles family of methods (ROCIS and PNO-ROCIS) and their slightly parameterized versions, [ROCIS/DFT and PNO-ROCIS/DFT].^48−50^ In this family of methods, one starts from a high-spin (HS) CSF and solves a CI problem with spin-adapted single CSFs. The attractive feature of ROCIS over methods of comparable computational complexity, such as TD-DFT, is that it treats the electron spin more rigorously. Thus, all ROCIS states are eigenfunctions of the total spin squared operator. This allows us to explicitly represent all *M*_S_ levels of each electronic state and their complex interactions through SOC including the states that, relative to the ground-state total spin *S*, have increased (*S* + 1) or decreased (*S* – 1) spin angular momentum. The latter spin flip states are, in fact, of crucial importance in order to compute even qualitatively correct spectra at the L- and M-edges. Thus, the ROCIS family of methods provides a realistic relativistic many-particle spectrum and at the same time is applicable to larger systems. The methodology has seen a number of successful applications, also to rather advanced spectroscopic methods such as L-edge XAS, RIXS,^51^, or XMCD and core resonant X-ray emission spectra (VtC-RXES).^51^ In particular, the PNO-ROCIS and PNO-ROCIS/DFT variants are readily applied to “real life” such as small peptides or surfaces. As discussed at length elsewhere,^49,50^ the same is not true for particle-hole-based methodologies that fail to properly span the final state manifold in cases with important multiplet effects.

Up to this point, an important limitation of the ROCIS method is that systems featuring an antiferromagnetically coupled ground state cannot be treated since the reference determinant is always assumed to be of the high-spin type. A high-spin determinant features a set of doubly occupied molecular orbitals (DOMOs) and a set of singly occupied MOs (SOMOs) in which all spins are aligned in parallel. Thus, for *n* SOMOs, one creates a spin eigenfunction with total spin . The case where not all unpaired electrons are spin-aligned is of very much higher complexity since a properly spin-adapted wave function is no longer a single determinant. This will be elaborated on in detail in further sections.

Given the exponentially increasing complexity of the computational problem, it is certainly tempting to simply disregard the antiferromagnetic coupling and assume that ferromagnetic spin alignment should work even in the case that the real system is antiferromagnetic given that the individual ions that are probed in the X-ray experiment are locally in a high-spin configuration. However, this is not the case. We have met cases in which ferromagnetic alignment of local spins leads to qualitatively wrong results. One such case is the Co L-edge XAS spectrum of Co_3_O_4_ where the failure of the standard high-spin ROCIS (HS-ROCIS)^52^ has in fact triggered the research efforts reported in this work.

Thus, for antiferromagnetic systems, one presently only has three choices: (1) to use an elaborate multireference wave function-based method, (2) disregard the antiferromagnetic coupling and align the local spins in parallel, or (3) break the spin symmetry and run XAS calculations off a broken symmetry (BS) determinant. The multireference option is rigorous but very quickly gets out of hand in terms of its computational complexity. Presently, treatments that incorporate dynamic electron correlation can, at most, treat two centers. Thus, there is a pressing need for further development in that area.

In the framework of density functional theory (DFT), time-dependent DFT (TD-DFT) is usually used on top of a BS-DFT solution. This practice has been proven instrumental in studying classes of molecular systems in the fields of inorganic chemistry and biochemistry.^16,17,23^ However, in systems with complex spin coupling situations, there are several possible broken-symmetry solutions, making it difficult to assign a given spin situation to the system.^17,23^ It should also be emphasized that BS-DFT gives only a crude representation of antiferromagnetic coupling and, like any particle-/hole-based theory, also fails to capture multiplet effects correctly.

Given the need for a low-cost method that properly treats multiplet effects and antiferromagnetic coupling, we set out to develop a version of ROCIS that can be applied to antiferromagnetically coupled ground states. In this case, one still bases the calculation on a single electronic configuration in which each orbital is either singly or doubly occupied (or empty), but the reference wave function is multideterminantal due to the coupling of the unpaired spins to a total spin that is less than the maximum spin (). Accomplishing this task purely algebraically is quite daunting as each individual coupling case creates a distinct set of configuration interactions (CI) that would need to be implemented. Thus, we have made use of the general infrastructure that the recently developed tree-based ICE algorithm^53,54^ offers and have developed a prototype approach that lets us generate all complex interactions between spin-coupled reference and excited CSFs on the fly. In the case of open-shell chemical systems, the choice of the reference CSF and the orbitals that comprise it is best addressed by solving the SCF problem of the recently developed CSF ROHF method.^55^ This ensures a proper and uniform description of the resonance CSF and the ROCIS excitation problem that is built upon.

We refer to the resulting method as “general spin” ROCIS (GS-ROCIS). In this nomenclature, the existing high-spin ROCIS (HS-ROCIS) is a special case of GS-ROCIS. Using that methodology, we are able to treat systems as complex as antiferromagnetic solids. The combination with the PNO concept developed earlier^50^ leads to PNO-GS-ROCIS, which consequently is a highly attractive, low-cost method to treat the spectroscopy of large systems with complex spin couplings.

## Theory

### Recapitulation of the High-Spin ROCIS Problem

The original formulation of ROCIS^48,49^ is based on a high-spin CSF reference. In this framework, upon defining a proper set of ROHF or DFT molecular orbitals, the ROCIS wave function is written as a linear combination of the reference (|Φ_0_⟩) and excited, spin-adapted CSFs (|Φ_*q*_^*p*^⟩) for a given total spin *S*
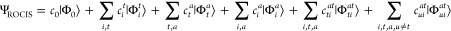
1

where the classes of excited CSFs are defined by the application of the singlet excitation operator , with  and  being the second quantized creation and annihilation operators, respectively. Here and throughout the text, we use the indices *i*, *j*, *k*, and *l* for doubly occupied molecular orbitals (DOMOs), *t*, *u*, *v*, and *w* for singly occupied molecular orbitals (SOMOs), *a*, *b*, *c*, and *d* for virtual orbitals (VMOs), and *p*, *q*, *r*, and *s* for generic molecular orbitals.

The expansion coefficients *c* are determined by the diagonalization of the Born–Oppenheimer Hamiltonian over the CSFs, with matrix elements given by
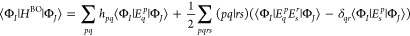
2where Φ_*I*_ and Φ_*J*_ represent arbitrary CSFs, and *h*_*pq*_ and (*pq*|*rs*) are the one- and two-electron integrals, respectively.

The key point in the construction of the Hamiltonian matrix is the calculation of matrix elements ⟨Φ_*I*_|*E*_*q*_^*p*^|Φ_*J*_⟩ and ⟨Φ_*I*_|*E*_*q*_^*p*^*E*_*s*_^*r*^|Φ_*J*_⟩, known as the one- and two-body coupling coefficients, respectively. In the high-spin ROCIS formulation, these elements can be obtained via the commutation rules of the *E*_*q*_^*p*^ operators, together with additional relations (eq 3) which reduce these matrix elements to a string of Kronecker deltas, as detailed in ref (48).
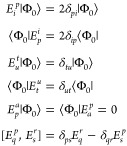
3

Being able to compute the ⟨Φ_*I*_|*E*_*q*_^*p*^|Φ_*J*_⟩ and ⟨Φ_*I*_|*E*_*q*_^*p*^*E*_*s*_^*r*^|Φ_*J*_⟩ matrix elements, the CI problem is solved in a direct CI fashion by means of a Davidson diagonalization routine.^56^

### Formulation of the General-Spin ROCIS Problem

**Figure 1:**
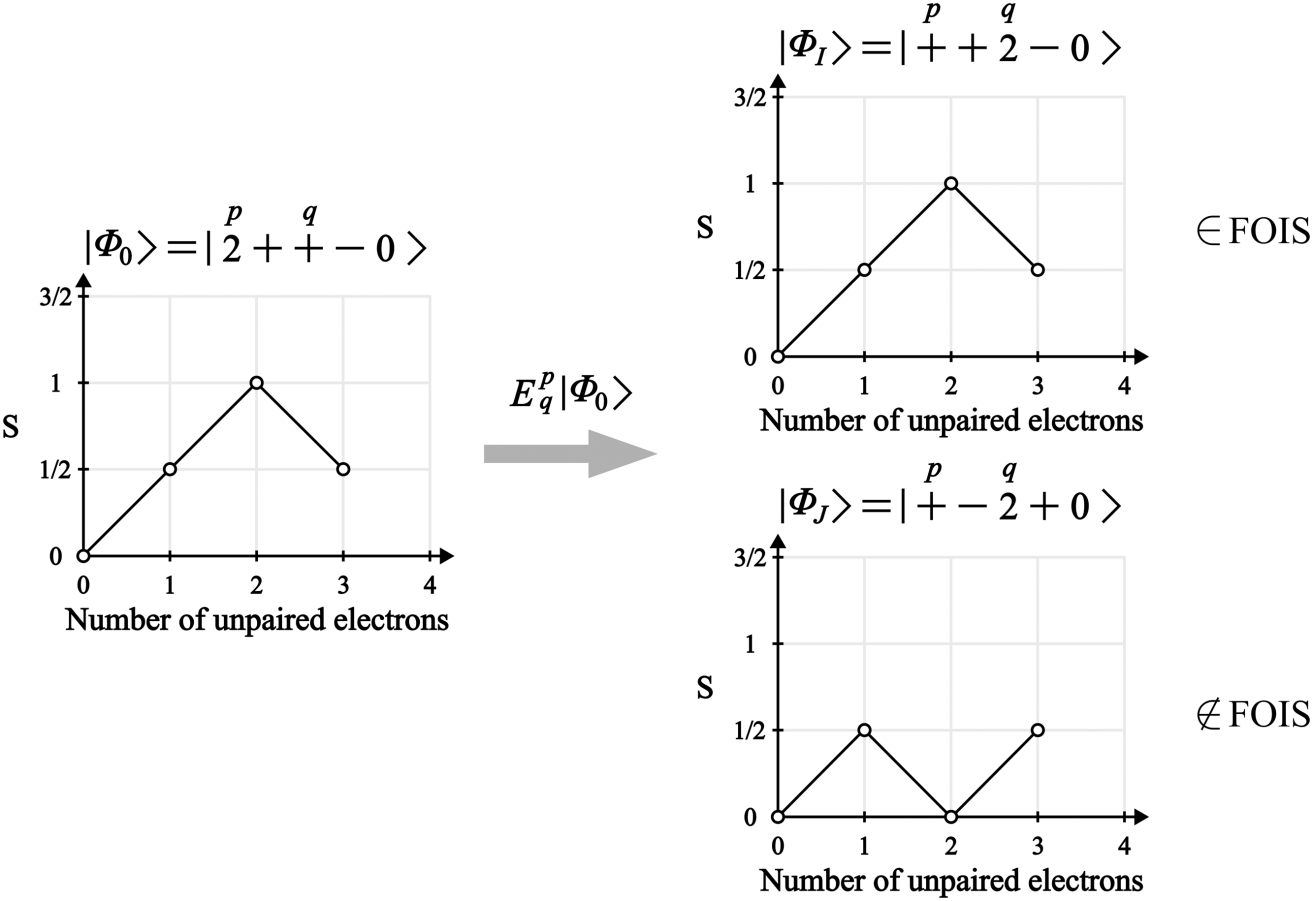
Application of the singlet excitation operator on a CSF generating two excited CSFs, of which only one interacts with the reference through *E*_*q*_^*p*^ and consequently part of the first-order interacting space (FOIS)^58^ of the GS-ROCIS problem.

We now formulate the ROCIS problem for an arbitrary reference CSF. For this, and throughout the description of the method, the CSFs are built following the genealogical spin coupling scheme,^57^ in which a given CSF is built via the sequential addition of electrons in a way that the spin coupling information on unpaired electron *u* is specified with respect to all other unpaired electrons *t* < *u*. The CSFs obtained this way are easily represented by a branching diagram (Figure 1), where a parallel coupling between consecutive spins is represented by an upward line and an antiparallel coupling by a downward line.

Differently from the original ROCIS formulation, the reference CSF can now have an arbitrary spin coupling situation from which a zeroth-order wave function is needed. This reference wave function is obtained from the recently developed CSF-ROHF method,^55^ which is able to perform ROHF-SCF calculation for a given CSF.

The excited CSFs are generated by the application of the singlet excitation operator on the reference (*E*_*q*_^*p*^|Φ_0_⟩). We note that the application of the *E*_*q*_^*p*^ operator on a single CSF can lead to more than one excited CSF, with a shared spatial part, but different spin-coupling patterns (Figure 1).

In order to generate only interacting excited CSFs from the application of the *E*_*q*_^*p*^ operator, we first generate the excited configuration by exciting an electron from orbital *p* to orbital *q* (if allowed from the occupation numbers of the involved orbitals). After the spatial excitation, the allowed spin couplings for the SOMOs in the generated CSFs are checked. Since *E*_*q*_^*p*^ is a singlet operator, it cannot change the total spin of the new CSFs, and due to the excitation occurring between orbitals *p* and *q*, all spin couplings outside the range of the excitation need to be the same as the parent CSF (Figure 1). Furthermore, the intermediate spin couplings between *p* and *q* must satisfy the triangle relation of the vector couplings. A detailed description of the branching diagram representation of the CSFs and the generation of excited CSFs by application of the *E*_*q*_^*p*^ can be found in ref (53).

With the strategy for the generation of the excited CSFs described, four excitation classes are defined according to the occupation number of the involved orbitals: DOMO to SOMO excitations (|Φ_*i*_^*t*^⟩), SOMO to VMO excitations (|Φ_*t*_^*a*^⟩), DOMO to VMO excitations (|Φ_*i*_^*a*^⟩), and DOMO to VMO excitations coupled with SOMO to SOMO excitations (|Φ_*ui*_^*at*^⟩), leading to the GS-ROCIS wave function
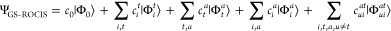
4

As shown in eq 2, to calculate the matrix elements of the BO-Hamiltonian, it is necessary to obtain the one- and two-body coupling coefficients. Differently from the HS-ROCIS, when dealing with an arbitrary reference CSF, it is, in general, no longer straightforwardly possible to reduce these coupling coefficients to sums of Kronecker deltas since the rules *E*_*u*_^*t*^|Φ_0_⟩ = δ_*tu*_|Φ_0_⟩ and ⟨Φ_0_|*E*_*t*_^*u*^ = δ_*ut*_⟨Φ_0_| no longer hold. Hence, a different strategy needs to be employed in calculating the coupling coefficients. We can, however, still employ the rules in eq 3 in evaluating elements in which all orbital indices refer to DOMOs and VMOs. This way, it is possible to formulate analytical expressions for all matrix elements of the BO-Hamiltonian (see the Supporting Information), where the only coupling coefficients needed to be computed involve at least one SOMO index.

**Figure 2:**
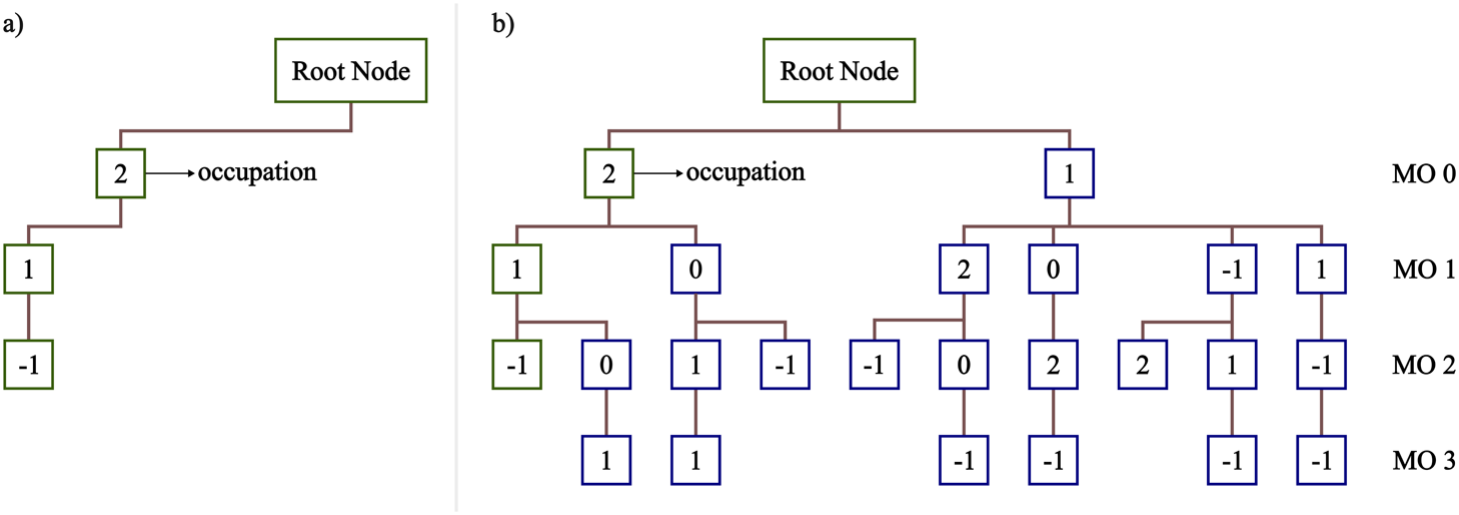
Schematic representation of the CSF tree data structure. (a) The CSF tree containing only the reference CSF. (b) The CSF tree containing all CSFs obtained from the ROCIS excitation classes.

Computation of the needed coupling coefficients is achieved by employing the same infrastructure used in the CSF-based ICE-CI method,^53,54^ where the CSFs are stored in a tree structure (Figure 2). For the GS-ROCIS problem, the CSF tree is constructed as follows: first, the reference CSF is introduced to the tree (Figure 2a), and then the respective *E*_*q*_^*p*^ operator for the excitation classes is applied to the reference CSF, generating all the possible excited CSFs, which are then inserted into the tree (Figure 2b). After all excited CSFs are inserted into the tree, both ⟨Φ_*I*_|*E*_*q*_^*p*^|Φ_*J*_⟩ and ⟨Φ_*I*_|*E*_*q*_^*p*^*E*_*s*_^*r*^|Φ_*J*_⟩ are directly calculated by a recursive tree walk algorithm which is discussed in detail in ref (53).

Since a GS-ROCIS calculation can involve hundreds of orbitals and even with efficient tree machinery, it would not be feasible to calculate all coupling coefficients necessary, making it necessary to employ a prototyping scheme, where only a reduced number of the coefficients need to be calculated.

The prototyping scheme employed follows from the realization that the values of the one- and two-body coupling coefficients are completely determined by the spin-coupling situation and the relative position of the *p*, *q*, *r*, and *s* orbital indices in relation to the SOMOs. This way, the recursive tree walk algorithm can be employed on a minimal orbital space able to represent all types of excited CSFs. Thus, the structure and size of the tree only depend on the number and coupling of singly occupied orbitals but are independent of system size. In fact, for the excitation classes used in the GS-ROCIS problem, the minimal required orbital space for prototyping consists of only two DOMOs, two VMOs, and the number of SOMOs in the reference CSF. The coupling coefficients calculated on the prototype space are stored and can be used whenever necessary in solving the CI problem.

We comment briefly on the strategy employed in the storage and retrieval of the prototype coupling coefficients. A given matrix element, ⟨Φ_*I*_|*E*_*q*_^*p*^|Φ_*J*_⟩, is stored with an address that is fully determined by the indices *p* and *q* in relation to the SOMOs and what kind of excitation is being performed on the bra CSF. For each of the 4 excitation classes, there exist two matrices: one that stores the addresses and another that stores the actual values of the coupling coefficients.

**Figure 3:**
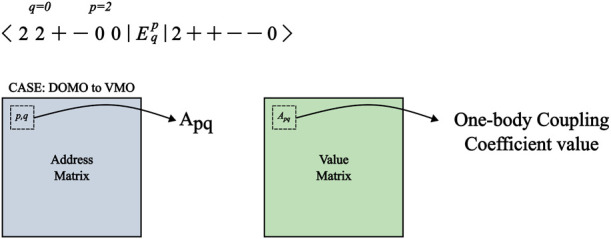
Schematic representation of the retrieval of a one-body coupling coefficient by the prototype scheme discussed in the text.

For example, in Figure 3, the ket CSF is obtained from the bra CSF by the excitation of *q* into *p*. Since there are no SOMOs before *q*, its relative index is 0, and since there are two SOMOs before *p*, its relative index is 2. The excitation case is a DOMO to VMO, which labels a matrix of coupling coefficients, the address of which is obtained by providing the relative indices of *p*, *q*.

**Figure 4:**
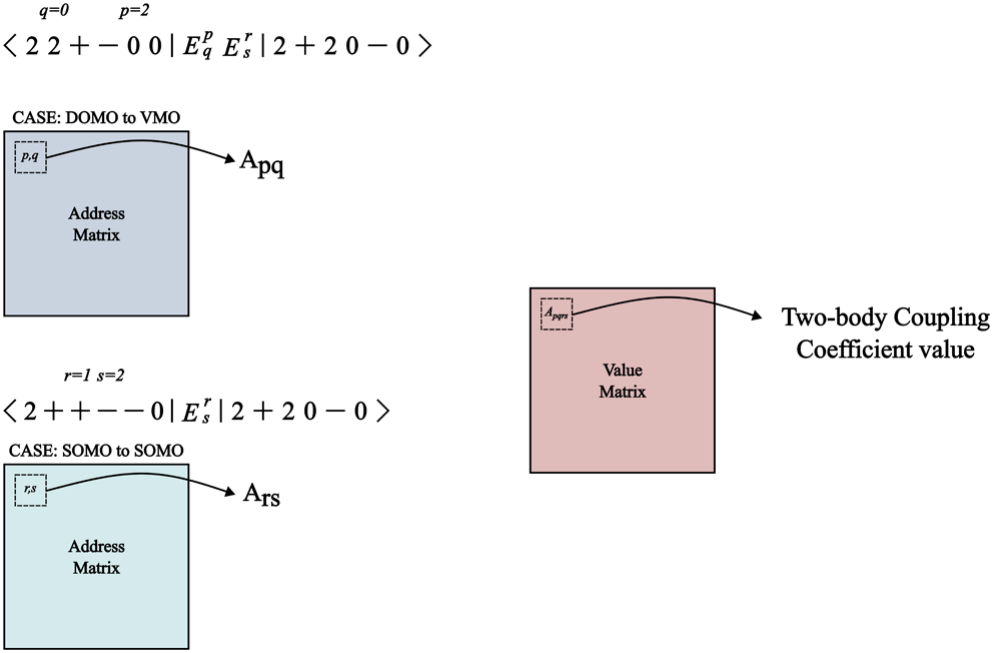
Schematic representation of the retrieval of a two-body coupling coefficient value by the prototype scheme discussed in the text.

For the two-body elements ⟨Φ_*I*_|*E*_*q*_^*p*^*E*_*s*_^*r*^|Φ_*J*_⟩, a similar procedure is used, but two steps are required in order to retrieve the coupling coefficients as shown in Figure 4. In the first step, *E*_*q*_^*p*^ is applied to a given CSF to obtain an intermediate CSF which is operated on by *E*_*s*_^*r*^ in order to obtain the target K CSF and the requested coupling coefficient.

As in the original ROCIS implementation,^48^ a Davidson diagonalization routine^56^ is employed in the determination of the eigenvalues and eigenvectors of the GS-ROCIS CI matrix, which requires the calculation of the σ-vector
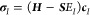
5where ***S*** is the overlap matrix of the expansion space, ***c***_*I*_ is the vector containing the coefficients of the CI expansion of state *I*, and *E*_*I*_ is the energy of this state. When solved by the appropriate vector ***c***_*I*_, **σ**_*I*_ has only vanishing elements. We note that by construction all CSFs employed in the presented method are orthonormal, resulting in the overlap matrix being simply the identity matrix.

Since in GS-ROCIS, we have four classes of excited CSFs and the reference CSF, the **σ**-vector is divided into 5 parts, which have to be calculated separately. The expressions for the elements of the sigma vector are obtained from the matrix elements of the BO-Hamiltonian and can be found in the Supporting Information. Below, we present how these elements are obtained.

Starting from the BO-Hamiltonian presented in eq 2 and applying the appropriate rules of the *E*_*q*_^*p*^ operators, we reduce any element to an expression involving only spatial integrals and coupling coefficients involving SOMO indices. As an example, for element ⟨Φ_0_|*H*|Φ_*i*_^*a*^⟩, we obtain
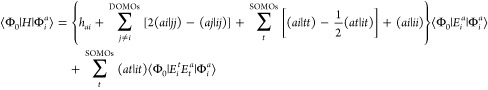
6

For convenience in evaluating the elements, we define three different types of Fock matrices, closed-shell, open-shell, and intermediate Fock matrices (*F*^C^, *F*^O^, and *F*^I^, respectively)
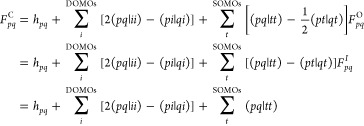
7

The closed-shell Fock matrix element from eq 7 arises from the ROHF Fock matrix as defined by Zerner and Edwards,^59^ when only the closed-shell part of the ROHF problem is considered. We emphasize that the Fock matrix formulation for the closed shell remains constant for all spin coupling situations that can be obtained from a CSF-ROHF^55^ calculation. The open-shell and intermediate Fock matrices are obtained from the closed-shell one by subtracting and adding , respectively.

Introducing the above-defined open-shell Fock into eq 6, we obtain
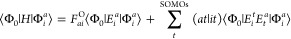
8where the coupling coefficients ⟨Φ_0_|*E*_*i*_^*a*^|Φ_*i*_^*a*^⟩ and ⟨Φ_0_|*E*_*i*_^*t*^*E*_*t*_^*a*^|Φ_*i*_^*a*^⟩ are computed by employing the prototyping scheme previously described.

### Transition Densities and Absorption Spectra

In order to calculate absorption spectra, a one-electron transition density between the states of interest is needed. In the second quantization, the transition density is defined as
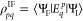
9where I and F are the initial and final states, respectively.

After the ROCIS problem is solved, the obtained states are linear combinations of the CSFs of the expansion space. Hence, the transition density between ROCIS states can be written as
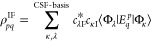
10

We can take advantage of the same prototyping scheme employed in the CI calculation for computation of the transition densities. This is achieved by determining the class of the matrix element ⟨Φ_λ_|*E*_*q*_^*p*^|Φ_κ_⟩ with respect to which type of excited CSFs are involved, from which the appropriate element can be read from the precomputed prototypes.

According to the general expression of light–mater interaction describing one photon processes, the intensity *I* of a given transition between states Ψ_I_ and Ψ_F_ depends on the transition moment , which in the second quantization is given by
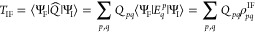
11where *Q*_*pq*_ is the one-electron integral of the respective one-electron operator *Q̂*, which in principle may represent several commonly used operators such as the electric dipole operator or the full field-matter interaction operator.^60^

### Computational Details

All calculations were performed in a development version of the ORCA 6.0 suite of programs.^61−65^ Molecular geometries were calculated at the DFT level of theory by employing the BP86 functional,^66,67^ together with Grimme’s dispersion correction^68−72^ with the triple-ζ Def2-TZVP basis set,^73^ together with the Def2/J auxiliary basis for the resolution of the identity (RI) approximation.^74^ The Ni chain geometry was fixed to local octahedral geometries,^75^ with only the hydrogen atoms being optimized at the same level of theory as the other geometry optimizations. The coordinates of all of the geometries used in this paper can be found in the Supporting Information.

All ROCIS calculations were performed using converged ROHF wave functions. For the systems where the reference wave function was not of the high spin case, the recently developed CSF-ROHF method^55^ was employed to obtain the ROHF solution of the desired CSF. The MRCI calculation was performed on selected reference CSFs in order to reproduce the GS-ROCIS problem.

BS-CIS calculations were performed using converged broken-symmetry unrestricted Hartree–Fock (UHF) wave functions as a reference.

For the Cu dimer, the Co_3_O_4_ spinel system, and the Ni chain, both ROCIS and BS-CIS calculations were performed with the Def2-SVP^73^ basis set. For the Ni dimer, ROCIS and MRCI calculations were performed with the Def2-TZVP basis set.

In all ROCIS calculations, the auxiliary basis sets Def2-SVP/C and Def2-TZVP/C were used for the RI approximation.

### Impact of Antiferromagnetic Coupling on Calculated Spectra: The Case of a Dicopper(II) System

**Figure 5:**
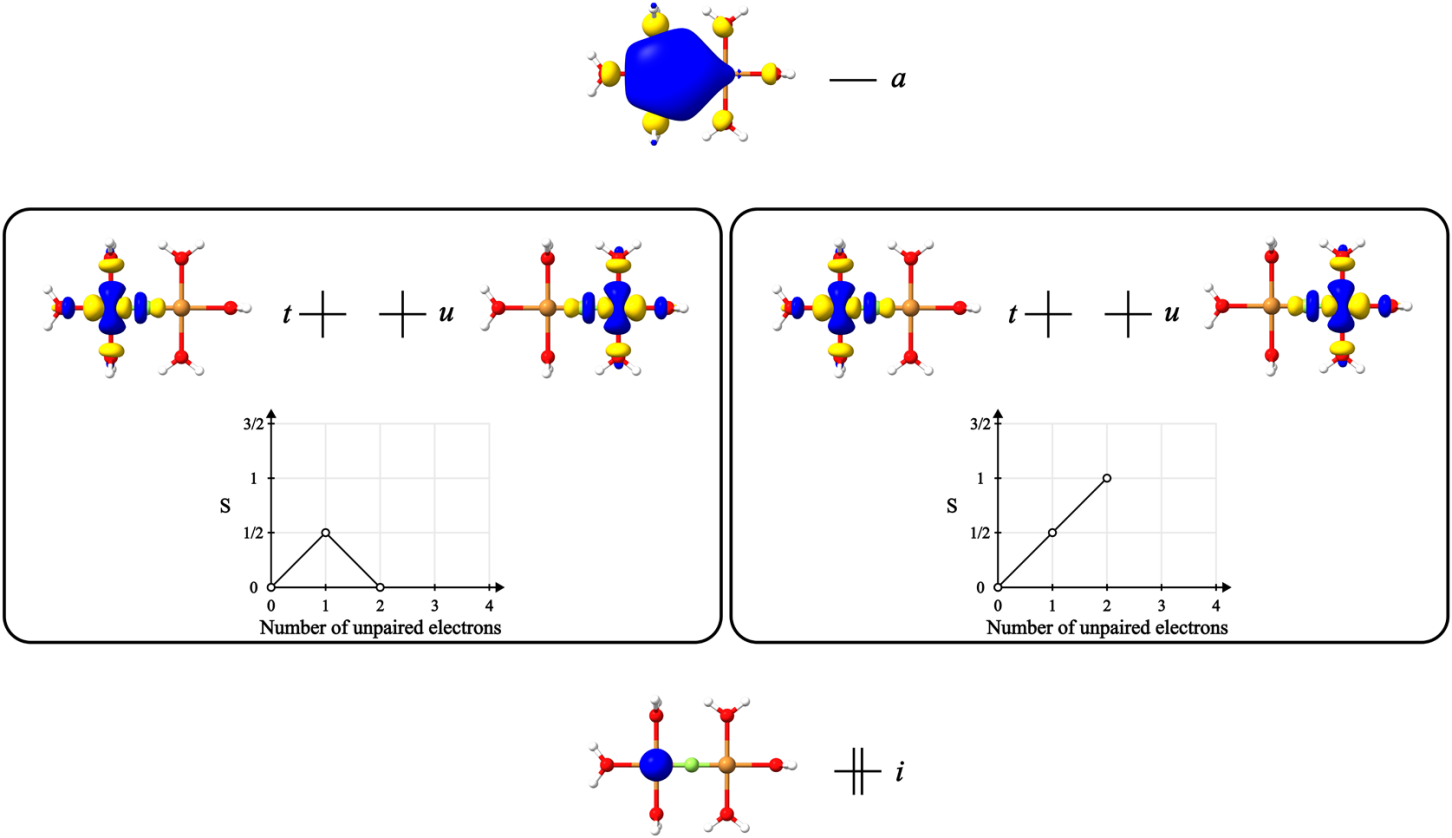
ROCIS space considered for the model system [Cu_2_(μ-F)(H_2_O)_6_]^3+^, consisting of the core 1s orbital of the Cu center, a single virtual orbital, and the two singly occupied orbitals obtained from converged ROHF calculations for a triplet and open-singlet states.

As an illustrative example of the differences between ROCIS calculations in different reference CSFs, we discuss the case of the model copper dimer [Cu_2_(μ-F)(H_2_O)_6_]^3+^ (Figure 5), where we consider triplet and open-singlet situations. The reference wave function for both situations was obtained by ROHF calculations, where for the open-singlet, the CSF-ROHF method^55^ was used to converge the SCF to the desired CSF, and for the triplet case, a high-spin solution was used. The resulting singly occupied orbitals are shown in Figure 5, together with the core orbital and the single virtual orbital used in this example. The SOMOs of the triplet were localized by a Pipek–Mezey localization scheme^76^ after convergence of the SCF in order to make the comparison between the two spin situations easier. The energy difference between the two ROHF solutions (*E*^HS-ROHF^ – *E*^CSF-ROHF^) is 146.3 cm^–1^ or 0.02 eV.

These reference wave functions are employed to construct a Cu K pre-edge excitation problem in which the excitation space consists of the core 1s orbital of one of the Cu centers as the sole donor orbital, the two singly occupied orbitals (SOMOs), and the lowest unoccupied orbital as the acceptor space.

**Figure 6:**
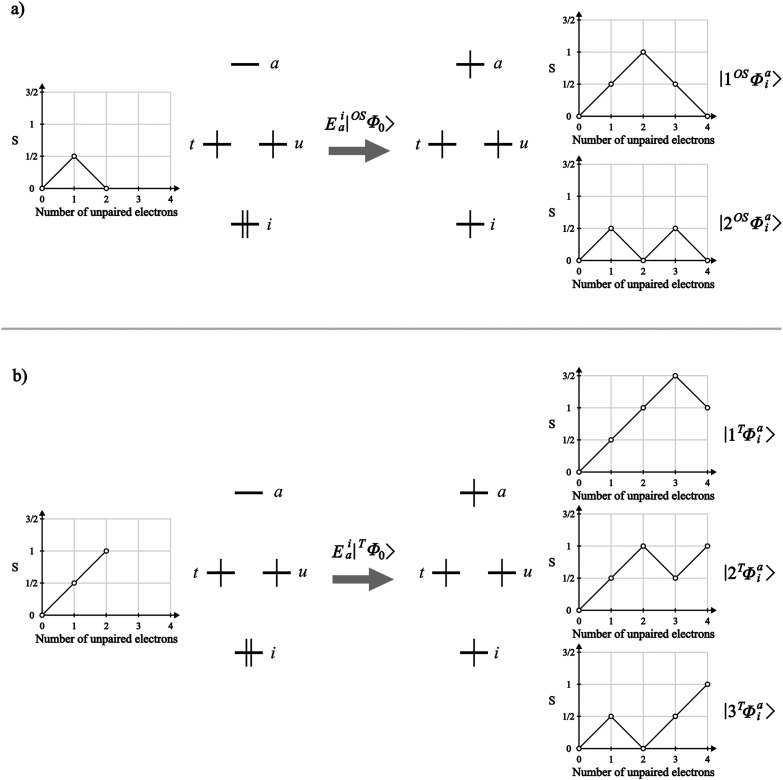
Branching diagrams for the DOMO → VMO excited CSFs from (a) the open-singlet reference CSF and (b) the triplet reference CSF.

We now proceed to formulate an example ROCIS calculation. In both cases (triplet and open-singlet), two excited CSFs of the type |Φ_*i*_^*t*^⟩ and two of the type |Φ_*ui*_^*at*^⟩ are generated from the reference, the branching diagram representation for these CSFs is the same as the reference since only the spatial part of the CSFs differs from the reference one. For the |Φ_*i*_^*a*^⟩ class, excitation from the core orbital into the virtual sphere creates two new unpaired electrons. The resulting 4 electrons are coupled to the appropriate multiplicity, which results in a different number of excited CSFs for each case (Figure 6). From the triplet ref (3), excited CSFs are obtained; from the open-singlet, only 2 excited CSFs arise. It is clear that the two situations described lead to distinct ROCIS problems, with different reference CSFs, excited CSFs, and dimensions of the CI expansion.

For comparison, one can perform a CIS calculation on a broken-symmetry reference (BS-CIS) as an approximation to the open-singlet situation. In this situation, the reference wave function is not a CSF but the broken-symmetry determinant , where η_*a*_ and η_*b*_ are the “magnetic” orbitals variationally relaxed in the SCF procedure. We reinforce that this determinant is not a spin eigenfunction of the system, while all CSFs used in the ROCIS method are spin-adapted. Single excitations from the broken-symmetry determinant do not produce the same space that ROCIS produces. Both |Φ_*i*_^*a*^⟩ and |Φ_*ui*_^*at*^⟩ classes of excitation are not captured in their entirety since the spin recouplings that are allowed in the |Φ_*i*_^*a*^⟩ excitation class of ROCIS would involve doubly excited determinants as would the |Φ_*ui*_^*at*^⟩ CSFs.

In fact, the restricted space used in this example for a BS-CIS calculation would result in only 2 excited states, resulting from excitations from the core to the virtual orbital. Since the BS reference determinant has the SOMOs as the highest occupied orbitals of the α and β set, it is not possible to have excitations directly into these orbitals.

We can also analyze how the different multiplicity of ROCIS problems can lead to different transition intensities for the excited states. Starting from the DOMO to SOMO excited CSFs, the transition densities between the reference CSF (|Φ_0_⟩) and the two resulting excited CSFs (|Φ_*i*_^*t*^⟩ and |Φ_*i*_^*u*^⟩) are the same, within a phase factor, in both triplet and open-singlet cases (Table 2). Since the intensity of the transition is dependent on the transition moment (eq 11), one can see that the one-electron integrals are the quantities that modulate the transition intensity. Hence, the different reference wave functions, from which the integrals are calculated, can result in different intensities for states dominated by the DOMO- to SOMO-excited CSFs.

Now going to the DOMO to VMO-excited CSFs (|Φ_*i*_^*a*^⟩), the picture changes. In this excitation class, as shown above, there is more than one CSF for each orbital excitation. The transition moments for the CSFs belonging to this class are presented in Table 1.where m_*ai*_^T^, m_*ai*_^OS^, and m_*ai*_^BS^ are the one-electron integrals of the transition operator for the triplet, open-singlet, and broken-symmetry references, respectively, and the CSF labels follow the ones in Figure 6.

**Table 1:** Transition Moments for the Triplet and Open-Singlet ROCIS and Also for the Broken-Symmetry CIS

triplet	open-singlet	BS-CIS
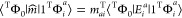	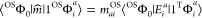	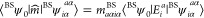
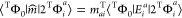	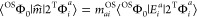	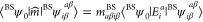
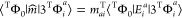		

Since the same integrals (within the multiplicity case) are used in the evaluation of the transition moments of the different CSFs, the difference in the magnitude of these is completely determined by the transition densities (Table 2), showing that the two different ROCIS problems can lead to different calculated spectra, not only due to being performed in different reference wave functions but also due to the intrinsic differences in the transition densities of the excited CSFs with the reference. As is evident from Table 2, the triplet reference leads to five distinct transitions with nonzero intensity and with an integrated intensity of 4.0 (au^2^). The open-singlet reference leads to only four “bright” transitions with the same integrated intensity of 4.0 (au^2^). In the case of the triplet reference, the most intense peak has an intensity of 1.33 (au^2^), while for the open singlet, a slightly higher intensity of 1.5 (au^2^) is expected.

**Table 2:** Non-zero Transition Densities between the Reference and Excited CSFs for the Triplet and Open-Singlet ROCIS Problems and the Sum of the Squares of the Given Values

triplet	open-singlet	BS-CIS
⟨^T^Φ_0_|*E*_*i*_^*t*^|^T^Φ_*i*_^*t*^⟩ = −1	⟨^OS^Φ_0_|*E*_*i*_^*t*^|^OS^Φ_*i*_^*t*^⟩ = −1	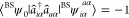
⟨^T^Φ_0_|*E*_*i*_^*u*^|^T^Φ_*i*_^*u*^⟩ = 1	⟨^OS^Φ_0_|*E*_*i*_^*u*^|^OS^Φ_*i*_^*u*^⟩ = −1	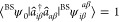
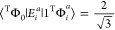	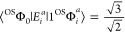	
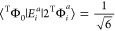	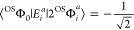	
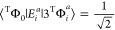		
sum(*m*^2^) = 4.0	sum(*m*^2^) = 4.0	sum(*m*^2^) = 2.0

Following these considerations, we can proceed numerically by solving the respective ROCIS and BS-CIS problems. For the open-singlet situation, 7 states are obtained, while for the triplet, there are 8 states in total, again reflecting the larger CI space as a consequence of the possible spin couplings of the |Φ_*i*_^*a*^⟩ excitation class. As previously stated, BS-CIS results in 2 excited states.

Comparing the excited-state energies (Table 3) obtained in both ROCIS calculations shows small differences in excited-state energies, which are a consequence of the two different CI problems being solved. The total spread of transition energies is nearly identical for the triplet and open-singlet cases, but there are variations in the intensity distribution that we will analyze below. BS-CIS gives only the equivalent of the |Φ_*i*_^*a*^⟩ excitations of ROCIS, not capturing the |Φ_*i*_^*t*^⟩ type of excitations.

**Table 3:** Excited States Obtained for the Excitation Problem Described Employing ROCIS on Both Triplet and Open-Singlet Situations^a^

state	ROCIS (triplet)	ROCIS (open-singlet)	BS-CIS
1	8922.46	8922.44	8951.49
2	8941.03	8941.10	8952.28
3	8951.71	8951.80	
4	8951.76	8951.92	
5	8951.92	8957.59	
6	8957.52	9009.90	
7	9009.74		
spread	87.3	87.5	0.79

a All energies are in eV.

The calculated oscillator strengths of the electric dipole operator for the transitions in both situations are listed in Table 4.

**Table 4:** Calculated Electric Dipole Oscillator Strength for the Different Transitions on Both Triplet and Open-Singlet ROCIS Problems as Well as the BS-CIS (*f*_ed_ Is Scaled by 10^6^)

Triplet			open-singlet			BS-CIS		
transition	*f*_ed_	composition	transition	*f*_ed_	composition	transition	*f*_ed_	composition
0 → 1	19.7	|Φ_*i*_^*t*^⟩	0 → 1	20.5	|Φ_*i*_^*t*^⟩			
0 → 2	0.0002	|Φ_*ui*_^*at*^⟩, |2Φ_*i*_^*a*^⟩	0 → 2	0.049	|Φ_*ui*_^*at*^⟩, |1Φ_*i*_^*a*^⟩			
0 → 3	1.80	|1Φ_*i*_^*a*^⟩, |2Φ_*i*_^*a*^⟩, |3Φ_*i*_^*a*^⟩	0 → 3	1.17	|1Φ_*i*_^*a*^⟩, |2Φ_*i*_^*a*^⟩	0 → 1	0.03	|ψ_*iα*_^*aα*^⟩
0 → 4	1.69	|1Φ_*i*_^*a*^⟩, |2Φ_*i*_^*a*^⟩, |3Φ_*i*_^*a*^⟩	0 → 4	2.32	|1Φ_*i*_^*a*^⟩, |2Φ_*i*_^*a*^⟩, |Φ_*ui*_^*at*^⟩	0 → 2	1.67	|ψ_*iβ*_^*aβ*^⟩
0 → 5	0.061	|1Φ_*i*_^*a*^⟩, |2Φ_*i*_^*a*^⟩, |3Φ_*i*_^*a*^⟩, |Φ_*ui*_^*at*^⟩						
0 → 6	10.2	|Φ_*i*_^*u*^⟩	0 → 5	7.87	|Φ_*i*_^*u*^⟩			
0 → 7	0.0059	|Φ_*ti*_^*au*^⟩, |2Φ_*i*_^*a*^⟩	0 → 6	0.012	|Φ_*ti*_^*au*^⟩, |1Φ_*i*_^*a*^⟩			

As seen from Table 4, the transitions with higher intensity on both the triplet and open-singlet references are to excited states composed of the |Φ_*i*_^*t*^⟩ and |Φ_*i*_^*u*^⟩, and the oscillator strengths are similar between the two different references due to the transition densities having the same magnitude, as seen in Table 2. Although the magnitude of the transition density is the same for both |Φ_*i*_^*t*^⟩ and |Φ_*i*_^*u*^⟩ CSFs, one observes that the former presents around half the intensity. This arises from the dipole integrals in the calculation of the transition moment. Since the transition, *i* → *t*, involves the SOMO on the same Cu center of the core electron being excited, the dipole integral of this transition is larger than the *i* → *u* transition, which involves the SOMO of the other Cu center.

Focusing on the transition to the second lowest excited state (0 → 2), we observe that in both triplet and open-singlet cases, the excited state involved in this transition is mainly constituted by the CSF |Φ_*ui*_^*at*^⟩. If these states were purely constituted of the mentioned CSFs, there would be no intensity to these transitions since |Φ_*ui*_^*at*^⟩ is a doubly excited CSF from the reference. All the observed intensity comes from a small mixing of the |2^T^Φ_*i*_^*a*^⟩ and |1^OS^Φ_*i*_^*a*^⟩ CSFs in the triplet and open-singlet cases, respectively. This mixing is given by the element
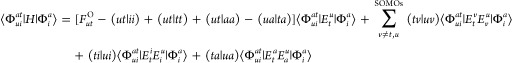
12

which shows that the mixing of the different CSFs depends on the values of the one- and two-body coupling coefficients since the spatial part of these CSFs is the same.

**Figure 7:**
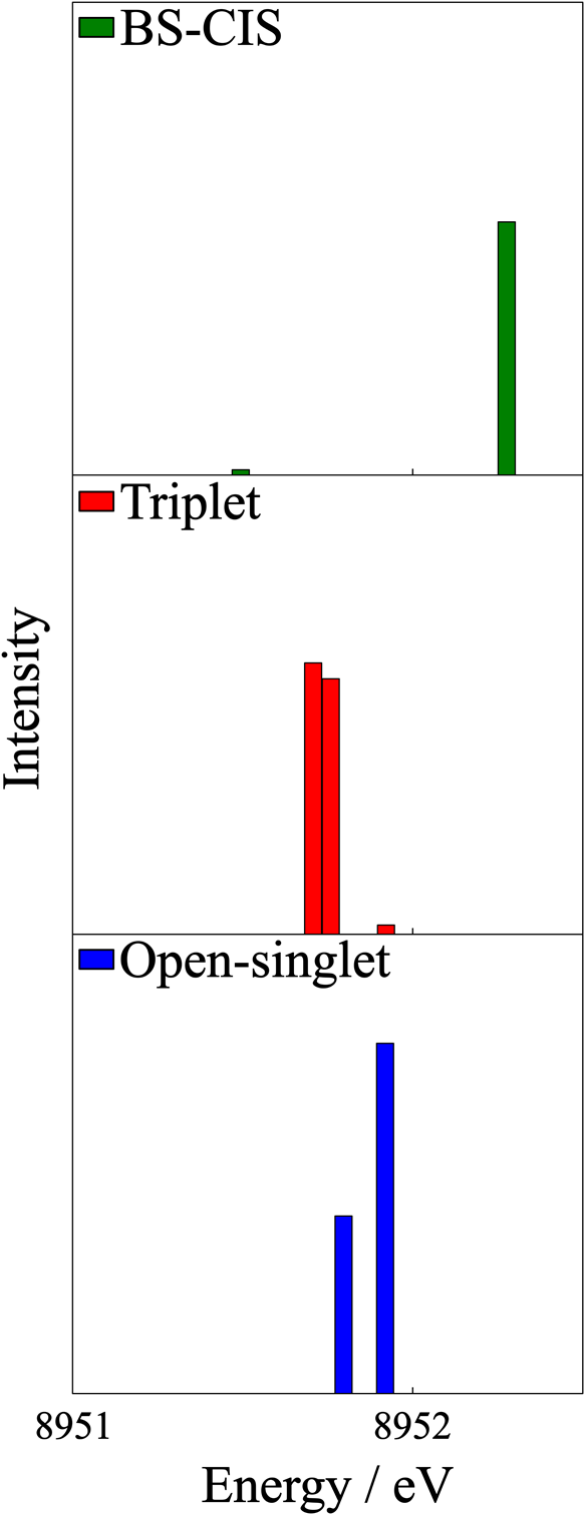
Stick plots of the dipole oscillator strengths for the |Φ_*i*_^*a*^⟩ excitations of triplet and open-singlet ROCIS as well as the equivalent transitions in BS-CIS. The relative intensities are preserved along the three plots.

From Figure 7 and Table 4, one observes that the oscillator strength of this transition is 2 orders of magnitude smaller in the triplet case in relation to the open singlet. Knowing that all intensity observed comes from the mixing of the |Φ_*i*_^*a*^⟩ CSFs into this state, we can compare the magnitudes of the transition densities of the triplet () and open-singlet () cases, showing that it is expected for this example that the transition in the open-singlet case to be more intense.

This small example shows that even on a simple system, employing ROCIS on a triplet or an open-singlet reference can lead to differences in calculated spectra due to different numbers of states in the respective CI problems, different spin couplings of the excited CSFs, and different transition densities of the CSFs. The CI procedure can then lead to mixings that can result in different intensities of the observed transitions, which depend on the spin coupling situation employed. Evidently, employing a BS-CIS method captures only a subset of the excitation problem due to this method not being able to generate the excited states that arise from doubly excited determinants. Hence, describing explicitly, the given AF excitation problem through the AF functionality of the GS-ROCIS seems to provide a robust computational strategy for the prediction of the core excited spectra of AF systems, which could be beneficial in the field of X-ray spectroscopy.

### Comparison to Higher-Level Methods and Experiment

**Figure 8:**
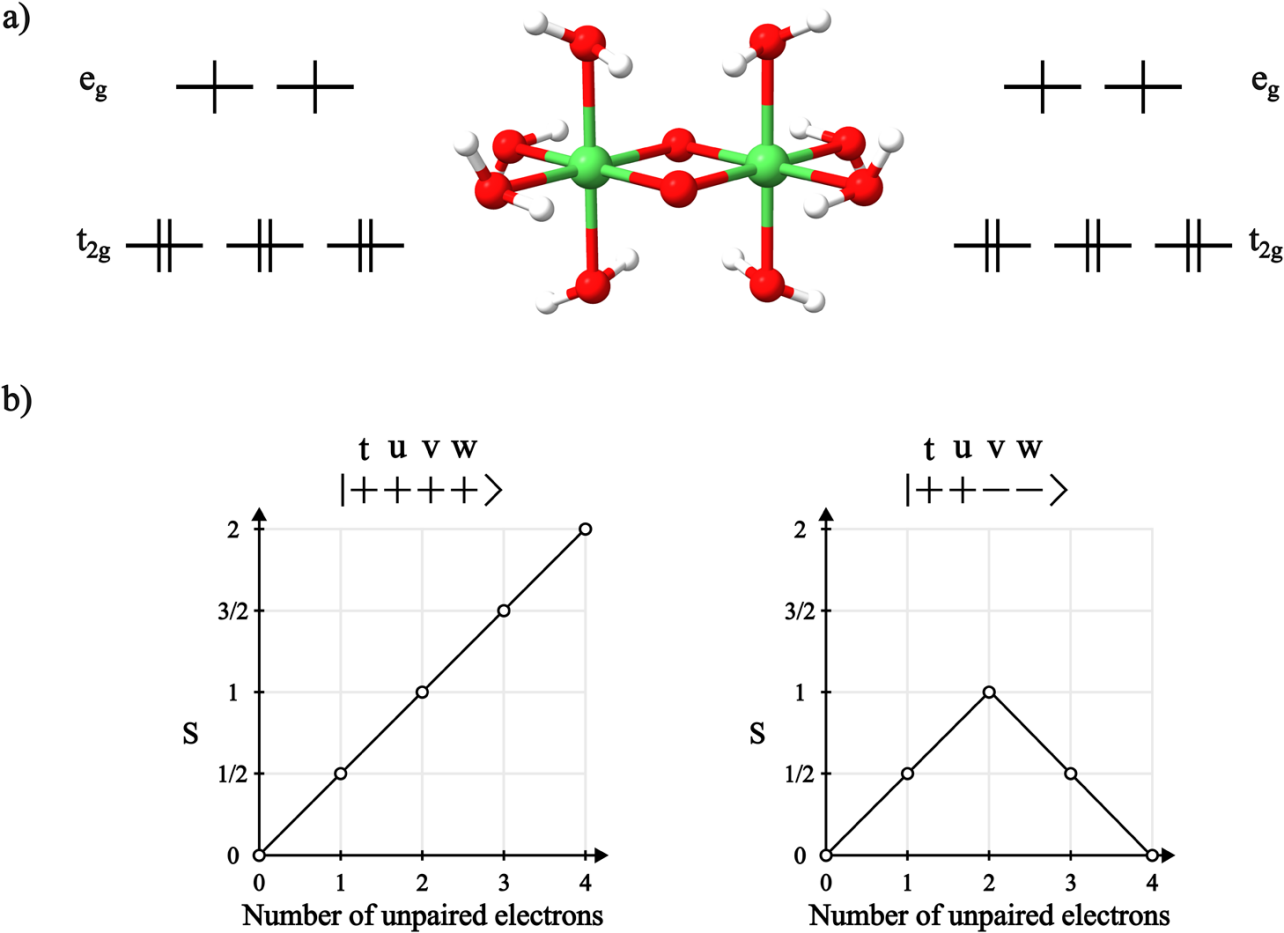
(a) Geometry and electronic structure of the Ni centers in [Ni_2_(μ-O_2_)(H_2_O)_8_]. (b) The branching diagram representing the reference CSFs with *S* = 2 (left) and *S* = 0 (right) on which the GS-ROCIS and MRCI calculations were performed. Also shown are the orbital labels used to distinguish the different SOMOs in Table 5.

The accuracy of the new GS-ROCIS was first tested in calculating the pre-edge of dimer [Ni_2_(μ-O_2_)(H_2_O)_8_] (Figure 8a) in the molecular geometry of the crystal of NiO, where the Ni centers have approximate octahedral symmetry. In *O*_*h*_ geometry, the Ni 3d orbitals split into two sets (*t*_2*g*_ and *e*_*g*_), where the *t*_2*g*_ orbitals are doubly occupied and the *e*_*g*_ orbitals are singly occupied, resulting in local *S* = 1 metal centers that can couple with each other in a ferromagnetic or antiferromagnetic fashion, resulting in total spins *S* = 2 and *S* = 0, respectively.

For this system, GS-ROCIS calculations were performed for both spin coupling cases. For the antiferromagnetic case, we also performed a MRCI tailored to include only excited configurations pertaining to the GS-ROCIS excitation space. This serves to check the correctness of the GS-ROCIS implementation and whether the slight methodological differences make substantial differences in the final results. In particular, the MRCI program always constructs and processes all possible spin couplings for a given configuration while, as explained above, GS-ROCIS applies the spin-traced excitation operator directly to the reference wave function, thus potentially creating fewer CSFs than the MRCI treatment.

The orbital excitation space consists of the 1s orbitals of the Ni centers as donor orbitals and the entire set of SOMOs and VMOs as acceptor orbitals from which the excited CSFs are constructed. The reference wave function was obtained by ROHF calculations for both the ferromagnetic and antiferromagnetic systems. In the ferromagnetic case, a high-spin ROHF calculation was performed; as for the antiferromagnetic case, the ROHF was converged to the CSF represented in the right branching diagram of Figure 8b, which is the one that more properly describes the antiferromagnetic coupling of the ground state. We reiterate, however, that a full description of antiferromagnetic coupling requires a multiconfigurational wave function. The CSF-ROHF solution is only an approximation to such a wave function that captures the leading spin-coupled configuration but misses the ionic components in the wave function that bring in the actual lowering of the lower-spin states.^55^

The excited states relevant to the pre-edge obtained by the calculations are listed in Table 5. As expected, these states are mainly constituted by |Φ_*i*_^*t*^⟩-type CSFs, where the Ni core 1s electron is excited to the *e*_*g*_ SOMOs. However, it is readily observable that the number of final states differs between the MRCI and the GS-ROCIS calculations.

**Table 5:** Composition and Energies (in eV) of the States in the Pre-edge Region of [Ni_2_(μ-O_2_)(H_2_O)_8_] Obtained with GS-ROCIS and MRCI^a^

	GS-ROCIS (S = 2)		GS-ROCIS (*S* = 0)		MRCI (*S* = 0)^b^	
state	composition	energy	composition	energy	composition	energy
1	47% |Φ_*i*_^*v*^[++++]⟩	8283.50	96% |Φ_*i*_^*t*^[++–−]⟩	8283.48	96% |Φ_*j*_^*v*^⟩	8431.52
	49% |Φ_*i*_^*w*^[++++]⟩					
2	47% |Φ_*j*_^*t*^[++++]⟩	8283.50	24% |Φ_*j*_^*v*^[++–−]⟩	8283.49	96% |Φ_*i*_^*t*^⟩	8431.52
	49% |Φ_*j*_^*u*^[++++]⟩		72% |Φ_*j*_^*v*^[+–+−]⟩			
3	49% |Φ_*i*_^*v*^[++++]⟩	8283.87	96% |Φ_*i*_^*u*^[++–−]⟩	8283.85	96% |Φ_*j*_^*w*^⟩	8431.89
	47% |Φ_*i*_^*w*^[++++]⟩					
4	49% |Φ_*j*_^*t*^[++++]⟩	8283.87	24% |Φ_*j*_^*w*^[++–−]⟩	8283.86	96% |Φ_*i*_^*u*^⟩	8431.93
	47% |Φ_*j*_^*u*^[++++]⟩		72% |Φ_*j*_^*w*^[+–+−]⟩			
5			72% |Φ_*j*_^*v*^[++–−]⟩	8286.01	96% |Φ_*j*_^*v*^⟩	8434.06
			24% |Φ_*j*_^*v*^[+–+−]⟩			
6			72% |Φ_*j*_^*w*^[++–−]⟩	8286.38	96% |Φ_*i*_^*t*^⟩	8434.06
			24% |Φ_*j*_^*w*^[+–+−]⟩			
7					96% |Φ_*j*_^*w*^⟩	8434.44
8					96% |Φ_*i*_^*u*^⟩	8434.47

a The indices *i* and *j* refer to the 1s core orbital of the distinct Ni centers. The indices *t*, *u*, *v*, and *w* refer to the SOMOs of the Ni centers as shown in Figure 8. In square brackets, the spin coupling situation of the excited CSF.

b The MRCI method implemented in ORCA uses configurations (CFG) as many-particle basis functions, where a single CFG contains all CSFs of the same orbital configuration. Hence, it is not possible to specify the individual spin coupling contributions of a given state.

In the MRCI (*S* = 0) calculation, 8 states are obtained that originate from the four possible DOMO to SOMO excitations (two for each Ni center). For all four excitations, there are two possible spin couplings thus resulting in 8 states. Of this manifold, only the 4 lowest energy ones are connected to the ground-state CSF and therefore potentially carry intensity.

By contrast, in the GS-ROCIS scheme for the *S* = 0, only 6 states arise. This is due to the restriction imposed by the direct application of the *E*_*q*_^*p*^ operator on the reference CSF, which prevents the creation of the excited CSF with [+–+−] spin coupling for the excitations on Ni defined as the “up” part of the branching diagram of Figure 8b. However, the absence of these, excited CSFs has no apparent impact on the calculated energies of the excited states. In addition, these missing CSFs do not contribute any intensity to the spectrum. This is an obvious result because the field-matter operator is a one-electron operator (that contains the operator *E*_*q*_^*p*^) and the additional CSFs, by construction, are not connected to the reference state by the action of *E*_*q*_^*p*^ on |0⟩.

Turning now to the *S* = 2 case, only four states are obtained. They have the same orbital origin as in the *S* = 0 case, but only a single spin coupling is possible. In this situation, the manifolds in the MRCI and GS-ROCIS methods are identical.

**Figure 9:**
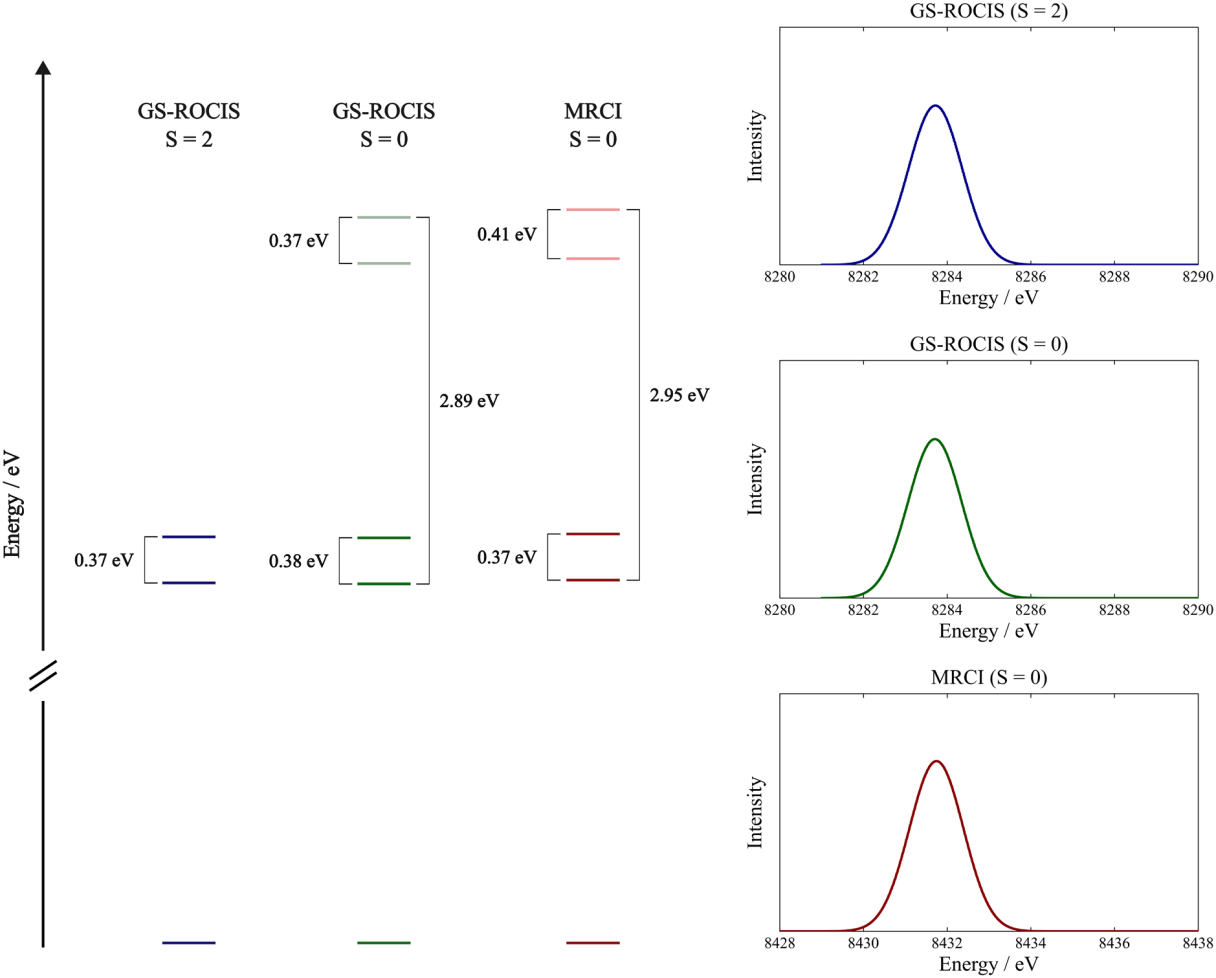
Energy differences between the lowest-lying core excited states of the nickel centers in the dimer [Ni_2_(μ-O_2_)(H_2_O)_8_] calculated using GS-ROCIS for the ferromagnetic coupling situation and GS-ROCIS and MRCI for the antiferromagnetic coupling situation. Also shown are the calculated absorption spectra obtained in the three calculations, where a Gaussian line broadening of 1.44 eV was employed on the oscillator strengths calculated with the full field-matter interaction operator.^60^

The calculated absorption spectrum obtained from the calculations is presented in Figure 9, where the relative energies of the excited states are also shown. Based on the discussion above, it becomes clear why the MRCI and GS-ROCIS calculations provide nearly identical spectra despite the fact that the MRCI calculation of the low-spin state features two additional CSFs in the excitation manifold.

**Figure 10:**
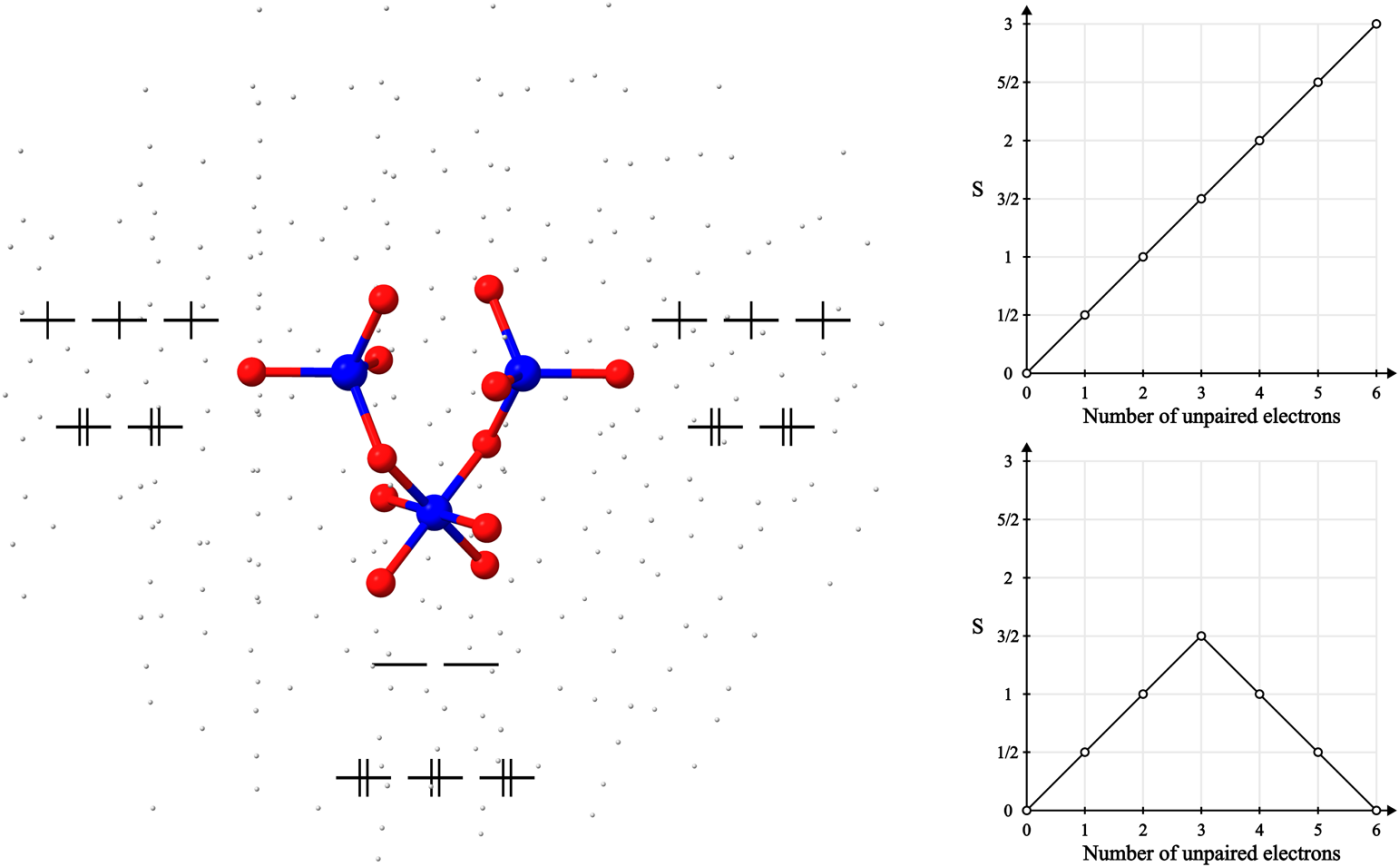
Quantum cluster consisting of two Co(II) centers and one Co(III) center, used for the calculation of the pre-edge spectrum of Co_3_O_4_ in an embedded cluster scheme and the branching diagrams representing the two spin coupling situations explored for the reference wave function in this paper.

Increasing the complexity of the system, we employed the GS-ROCIS method in the calculation of the pre-edge absorption spectrum of Co_3_O_4_ in the spinel crystal structure by using an embedded cluster approach in order to include the effects of the extended solid structure. In the spinel structure, Co_3_O_4_ consists of one Co(III) center with approximate octahedral symmetry and two Co(II) centers with approximate tetrahedral symmetry (Figure 10). The Co(III) center is low-spin with local *S* = 0, and the Co(II) centers have local .

For the purposes of this paper, a quantum cluster (QC) consisting of a minimal subunit was employed. This QC was embedded in a point charge field with a Hartree–Fock layer in order to neutralize the total charge of the system.

The pre-edge spectrum was calculated using GS-ROCIS on the QC in both ferromagnetic and antiferromagnetic coupling scenarios, where the two Co(II) centers couple, resulting in a total *S* = 3 and *S* = 0, respectively. For the *S* = 0 system, the reference wave function was obtained by converging a CSF-ROHF calculation to the CSF represented by the branching diagram shown in Figure 10. For comparison, an unrestricted CIS calculation on top of a broken-symmetry determinant was also employed for calculating the pre-edge of the antiferromagnetic situation.

**Figure 11:**
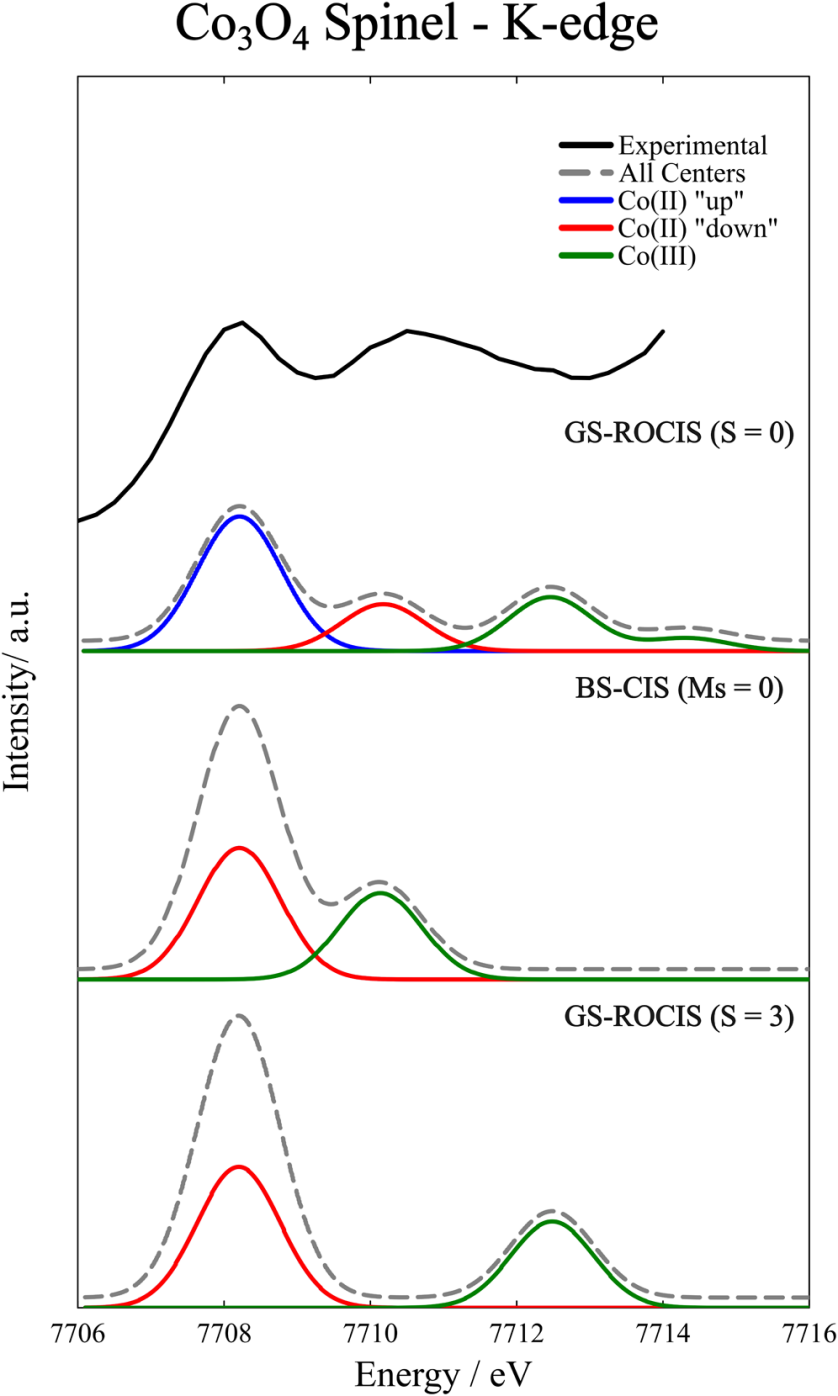
Experimental^77^ and calculated pre-edge spectrum of Co_3_O_4_ in the spinel crystal structure. Oscillator strengths were calculated with the full field-matter interaction operator.^60^ For all calculated spectra, a Gaussian broadening of 1.33 eV was applied. Both GS-ROCIS-calculated spectra were shifted by 36.1 eV, and the BS-CIS spectrum was shifted by 33.6 eV.

The spectra calculated with GS-ROCIS and BS-CIS are shown in Figure 11, together with the experimental K β-detected HERFD XAS spectrum taken from ref (77).

In all cases, we observe distinct bands for the excitation of the Co(III) center and of the Co(II) centers. The excitations in the Co(III) center are mainly from the 1s core to the empty 3d orbitals. In both BS-CIS and GS-ROCIS for the *S* = 3 system, these excitations give rise to a single band. When going to the GS-ROCIS calculation for the *S* = 0 system, a splitting of this band is observed. This splitting results from the number of spin recouplings that arise from the excitation of the core to an empty orbital, which is larger in the case of the antiferromagnetically coupled system in a way similar to the observed splitting in the Ni dimer analyzed in the previous section.

Turning now to the Co(II) center, all calculations produce bands with energies lower than that for the Co(III) center. The most notable difference between the calculations is the additional band obtained in the GS-ROCIS calculation for the *S* = 0 system. Both bands result from excitations of the core 1s to the SOMOs of the Co(II); however, these DOMO to SOMO excitations are not equivalent between the centers when we have a branching diagram like the one in Figure 10 that shows direction reversal while traversing the graph, indicating antiferromagnetic coupling. These excitations, when occurring in the “up” metal center, do not allow for spin-recouplings in the “down” center since these are outside the range of the excitation operator *E*_*q*_^*p*^, resulting in fewer excited CSFs compared to the situation when the excitations occur on the “down” center, where spin recouplings on the “up” center are allowed. These extra excited CSFs mix in the CI procedure and result in a shift in the energy of the excited states observed in the pre-edge.

The obtained results indicate that the correct description of the magnetic coupling between metal centers can lead to differences in the calculated pre-edge spectra. While in the case of Co_3_O_4_, this result shows up in an intriguing way, one still needs to validate the conclusion against spin-sensitive X-ray spectroscopies (L-, M-edges, XMCD). As the description of these spectroscopies requires the explicit treatment of spin–orbit coupling, such a study is outside the scope of this paper.

We remark that GS-ROCIS does not explicitly include dynamic electron correlation, which limits its accuracy. GS-ROCIS also does not include double excitations, which affect the calculation of ligand-to-metal and metal-to-ligand charge transfer excitations.

### Performance Comparison to the Original ROCIS Implementation

**Figure 12:**
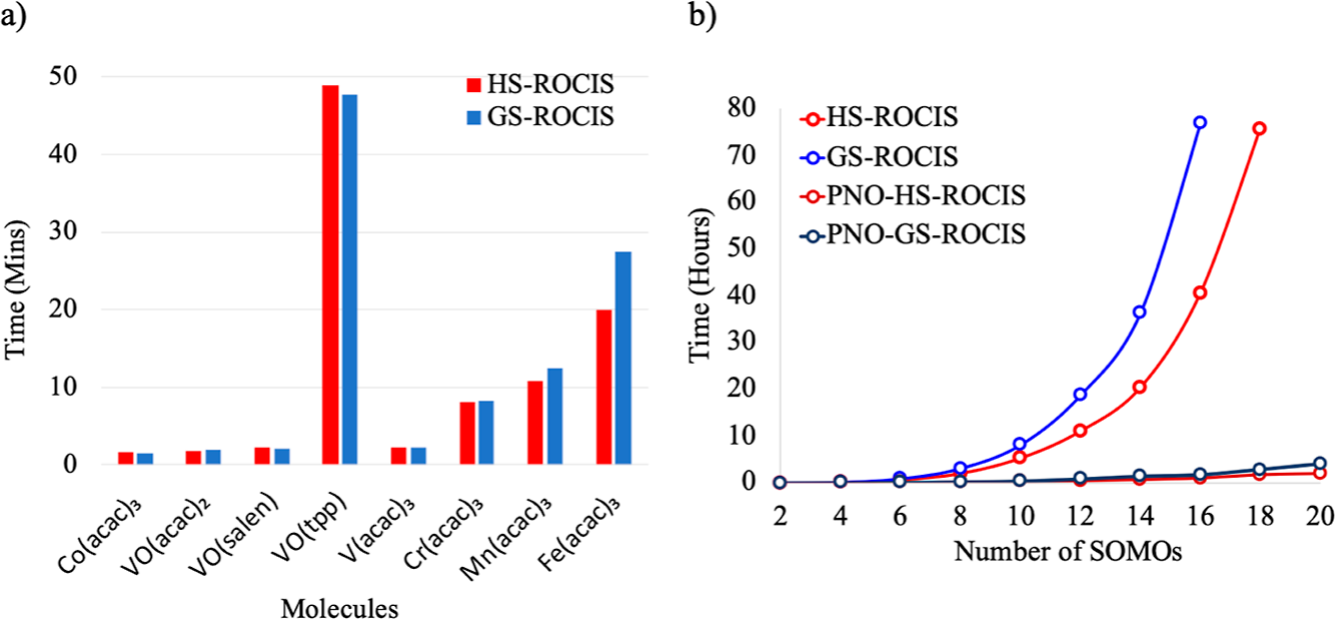
(a) Time comparison between HS-ROCIS and GS-ROCIS on a series of transition metal complexes. (b) Scaling comparison between both codes on an increasing nickel chain.

In the final section of this study, we evaluate the performance of the new GS-ROCIS in an effort to investigate the actual cost of having an ROCIS family of methods that are able to treat the core excitation problem of systems with arbitrary spin couplings. For this purpose, GS-ROCIS was compared with the original HS-ROCIS method in two ways. First, we ran calculations of both codes in a series of representative transition metal complexes (Figure S1), with different number of singly occupied orbitals and different number of basis functions. The comparison between the GS- and HS-ROCIS timings on these complexes is shown in Figure 12a.

We observe that both codes perform equally with an increasing number of basis functions for a given number of singly occupied orbitals, as seen in the series of vanadyl complexes. The main difference arises when increasing the number of SOMOs, where the GS-ROCIS code performs circa 1.3 times slower for the [Fe(acac)_3_] complex (5 SOMOs, ).

**Figure 13:**
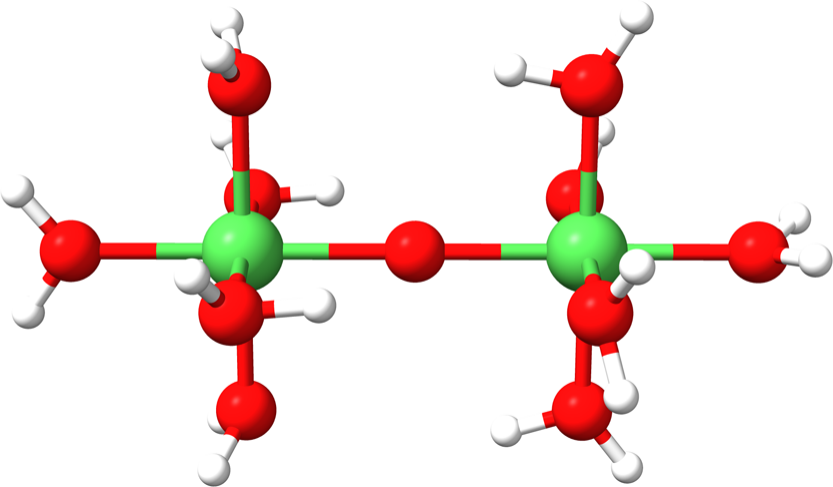
Nickel chain fragment used in the scaling study of GS-ROCIS.

In order to investigate further the computation time dependency on the number of SOMOs of the system, a series of calculations were also performed on a Ni(H_2_O)_5_[Ni(O)(H_2_O)_4_]_*n*_(H_2_O) chain (Figure 13), where n was gradually increased. Each nickel added to the chain was coupled ferromagnetically with the previous one in order for the system to be properly described by both codes. The total ROCIS calculation time for each chain size is shown in Figure 12b, where we see that up to 16 SOMOs (8 nickels in the chain), the GS-ROCIS is still less than 2 times slower than the original HS-ROCIS implementation. This implies that the two canonical methods could be in principle interchangeable without noticeable loss of performance. Switching to the PNO versions of the two methods provides the expected linear scaling with the system size, leading to orders of magnitude of acceleration (e.g., not less than a factor of 40 in the case of 16 SOMOs).

All comparisons were performed in an Intel cluster using 8 cores and 10 GB of RAM per core.

## Conclusions

In this work, we have presented a general spin ROCIS method that allows the ROCIS calculation to be performed on top of a reference CSF with an arbitrary spin coupling situation. This leads to a generalization of our previously developed ROCIS methods, while it provides access to the spectral computation of antiferromagnetically coupled chemical systems. The method is readily connected to the PNO-inspired truncation of the original HS canonical ROCIS and ROCIS/DFT methods formulating the PNO-GS-ROCIS variant of methods. By studying a model dicopper system, it was shown that GS-ROCIS, when applied to the ferromagnetic and antiferromagnetic coupling situations of this system, produces sets of excited CSFs that differ considerably in terms of their number and the described spin-coupling situations. Hence, the GS-ROCIS solution of the ferro- and antiferromagnetic systems leads to a different intensity mechanism of a given excitation problem. At the same time, it was shown that BS-CIS can describe only a subset of the excitation problem of interest that may or may not agree with the GS-ROCIS solutions.

The performance of the new GS-ROCIS method was further investigated in a dinickel system, where the spin coupling situation is more complex than that of the dicopper system. By comparison of the results with a MRCI calculation tailored to reproduce the ROCIS excitation space, it was demonstrated that GS-ROCIS is capable of calculating core excited states with similar accuracy. GS-ROCIS was also applied to the mixed valence system Co_3_O_4_ in the spinel structure, where it was further demonstrated how the correct treatment of the magnetic coupling between the Co(II) metal centers leads to differences in the calculated pre-edge spectra.

We believe that the developed GS-ROCIS and PNO-GS-ROCIS family of methods takes the next step forward in the field of wave function-based calculation of X-ray spectra since it allows one to tackle a variety of chemical systems possessing closed-shell and open-shell as well as arbitrary spin ground states. In addition, large systems can be treated with high efficiency and on modest hardware. Research in our laboratories is ongoing in order to further explore the abilities of the GS-ROCIS to complex polymetallic systems in a variety of X-ray spectroscopies. Our immediate goals are focused on applying the described GS-ROCIS in “real life” systems and also improving the method of electron correlation treatment.

## Supporting Information Available

All molecular geometries used in this paper, sample inputs, and calculated pre-edge spectra of the molecular test set used in Figure 12a (PDF)

## References

[ref1] de Groot F.; Kotani A. Core Level Spectroscopy of Solids; CRC Press: Boca Raton, 2008.

[ref2] Hadt R. G.; Hayes D.; Brodsky C. N.; Ullman A. M.; Casa D. M.; Upton M. H.; Nocera D. G.; Chen L. X. X-Ray Spectroscopic Characterization of Co(IV) and Metal–Metal Interactions in Co_4_O_4_: Electronic Structure Contributions to the Formation of High-Valent States Relevant to the Oxygen Evolution Reaction. J. Am. Chem. Soc. 2016, 138 (34), 11017–11030. 10.1021/jacs.6b04663. 27515121

[ref3] Haldrup K.; Gawelda W.; Abela R.; Alonso-Mori R.; Bergmann U.; Bordage A.; Cammarata M.; Canton S. E.; Dohn A. O.; van Driel T. B.; et al. Observing Solvation Dynamics with Simultaneous Femtosecond X-Ray Emission Spectroscopy and X-Ray Scattering. J. Phys. Chem. B 2016, 120 (6), 1158–1168. 10.1021/acs.jpcb.5b12471. 26783685

[ref4] Hirsch O.; Kvashnina K.; Willa C.; Koziej D. Hard X-Ray Photon-in Photon-out Spectroscopy as a Probe of the Temperature-Induced Delocalization of Electrons in Nanoscale Semiconductors. Chem. Mater. 2017, 29 (4), 1461–1466. 10.1021/acs.chemmater.6b05218.

[ref5] Kim T.; Song B.; Lunt A. J. G.; Cibin G.; Dent A. J.; Lu L.; Korsunsky A. M. Operando X-Ray Absorption Spectroscopy Study of Atomic Phase Reversibility with Wavelet Transform in the Lithium-Rich Manganese Based Oxide Cathode. Chem. Mater. 2016, 28 (12), 4191–4203. 10.1021/acs.chemmater.6b00522.

[ref6] Kowalska J. K.; Hahn A. W.; Albers A.; Schiewer C. E.; Bjornsson R.; Lima F. A.; Meyer F.; DeBeer S. X-Ray Absorption and Emission Spectroscopic Studies of [L_2_Fe_2_S_2_]_n_ Model Complexes: Implications for the Experimental Evaluation of Redox States in Iron–Sulfur Clusters. Inorg. Chem. 2016, 55 (9), 4485–4497. 10.1021/acs.inorgchem.6b00295. 27097289 PMC5108557

[ref7] Siebel A.; Gorlin Y.; Durst J.; Proux O.; Hasché F.; Tromp M.; Gasteiger H. A. Identification of Catalyst Structure during the Hydrogen Oxidation Reaction in an Operating PEM Fuel Cell. ACS Catal. 2016, 6 (11), 7326–7334. 10.1021/acscatal.6b02157.

[ref8] Yan J. J.; Gonzales M. A.; Mascharak P. K.; Hedman B.; Hodgson K. O.; Solomon E. I. L-Edge X-Ray Absorption Spectroscopic Investigation of {FeNO}_6_: Delocalization vs Antiferromagnetic Coupling. J. Am. Chem. Soc. 2017, 139 (3), 1215–1225. 10.1021/jacs.6b11260. 28006897 PMC5322818

[ref9] Henthorn J. T.; Arias R. J.; Koroidov S.; Kroll T.; Sokaras D.; Bergmann U.; Rees D. C.; DeBeer S. Localized Electronic Structure of Nitrogenase FeMoco Revealed by Selenium K-Edge High Resolution X-Ray Absorption Spectroscopy. J. Am. Chem. Soc. 2019, 141 (34), 13676–13688. 10.1021/jacs.9b06988. 31356071 PMC6716209

[ref10] Lassalle-Kaiser B.; Boron T. T. I.; Krewald V.; Kern J.; Beckwith M. A.; Delgado-Jaime M. U.; Schroeder H.; Alonso-Mori R.; Nordlund D.; Weng T.-C.; et al. Experimental and Computational X-Ray Emission Spectroscopy as a Direct Probe of Protonation States in Oxo-Bridged MnIV Dimers Relevant to Redox-Active Metalloproteins. Inorg. Chem. 2013, 52 (22), 12915–12922. 10.1021/ic400821g. 24161081 PMC3867288

[ref11] Kowalska J.; DeBeer S. The Role of X-Ray Spectroscopy in Understanding the Geometric and Electronic Structure of Nitrogenase. Biochim. Biophys. Acta, Mol. Cell Res. 2015, 1853 (6), 1406–1415. 10.1016/j.bbamcr.2014.11.027. 25486459

[ref12] Cutsail III G. E.; DeBeer S. Challenges and Opportunities for Applications of Advanced X-Ray Spectroscopy in Catalysis Research. ACS Catal. 2022, 12 (10), 5864–5886. 10.1021/acscatal.2c01016.

[ref13] Bauer M. HERFD-XAS and Valence-to-Core-XES: New Tools to Push the Limits in Research with Hard X-Rays?. Phys. Chem. Chem. Phys. 2014, 16 (27), 13827–13837. 10.1039/C4CP00904E. 24905791

[ref14] Zimmermann P.; Peredkov S.; Abdala P. M.; DeBeer S.; Tromp M.; Müller C.; van Bokhoven J. A. Modern X-Ray Spectroscopy: XAS and XES in the Laboratory. Coord. Chem. Rev. 2020, 423, 213466 10.1016/j.ccr.2020.213466.

[ref15] Pollock C. J.; DeBeer S. Insights into the Geometric and Electronic Structure of Transition Metal Centers from Valence-to-Core X-Ray Emission Spectroscopy. Acc. Chem. Res. 2015, 48 (11), 2967–2975. 10.1021/acs.accounts.5b00309. 26401686

[ref16] Krewald V.; Lassalle-Kaiser B.; Boron T. T. I.; Pollock C. J.; Kern J.; Beckwith M. A.; Yachandra V. K.; Pecoraro V. L.; Yano J.; Neese F.; et al. The Protonation States of Oxo-Bridged MnIV Dimers Resolved by Experimental and Computational Mn K Pre-Edge X-Ray Absorption Spectroscopy. Inorg. Chem. 2013, 52 (22), 12904–12914. 10.1021/ic4008203. 24161030 PMC3911776

[ref17] Krewald V.; Retegan M.; Cox N.; Messinger J.; Lubitz W.; DeBeer S.; Neese F.; Pantazis D. A. Metal Oxidation States in Biological Water Splitting. Chem. Sci. 2015, 6 (3), 1676–1695. 10.1039/C4SC03720K. 29308133 PMC5639794

[ref18] Beckwith M. A.; Ames W.; Vila F. D.; Krewald V.; Pantazis D. A.; Mantel C.; Pécaut J.; Gennari M.; Duboc C.; Collomb M.-N.; et al. How Accurately Can Extended X-Ray Absorption Spectra Be Predicted from First Principles? Implications for Modeling the Oxygen-Evolving Complex in Photosystem II. J. Am. Chem. Soc. 2015, 137 (40), 12815–12834. 10.1021/jacs.5b00783. 26352328

[ref19] Yano J.; Yachandra V. Mn4Ca Cluster in Photosynthesis: Where and How Water Is Oxidized to Dioxygen. Chem. Rev. 2014, 114 (8), 4175–4205. 10.1021/cr4004874. 24684576 PMC4002066

[ref20] Vinyard D. J.; Ananyev G. M.; Charles Dismukes G. Photosystem II: The Reaction Center of Oxygenic Photosynthesis. Annu. Rev. Biochem. 2013, 82, 577–606. 10.1146/annurev-biochem-070511-100425. 23527694

[ref21] Schuth N.; Gehring H.; Horn B.; Holze P.; Kositzki R.; Schrapers P.; Limberg C.; Haumann M. Biomimetic Mono- and Dinuclear Ni(I) and Ni(II) Complexes Studied by X-Ray Absorption and Emission Spectroscopy and Quantum Chemical Calculations. J. Phys.:Conf. Ser. 2016, 712 (1), 012134 10.1088/1742-6596/712/1/012134.

[ref22] Chrysina M.; Drosou M.; Castillo R. G.; Reus M.; Neese F.; Krewald V.; Pantazis D. A.; DeBeer S. Nature of S-States in the Oxygen-Evolving Complex Resolved by High-Energy Resolution Fluorescence Detected X-Ray Absorption Spectroscopy. J. Am. Chem. Soc. 2023, 145 (47), 25579–25594. 10.1021/jacs.3c06046. 37970825 PMC10690802

[ref23] Bjornsson R.; Lima F. A.; Spatzal T.; Weyhermüller T.; Glatzel P.; Bill E.; Einsle O.; Neese F.; DeBeer S. Identification of a Spin-Coupled Mo(III) in the Nitrogenase Iron–Molybdenum Cofactor. Chem. Sci. 2014, 5 (8), 3096–3103. 10.1039/C4SC00337C.

[ref24] Lukashuk L.; Föttinger K.; Kolar E.; Rameshan C.; Teschner D.; Hävecker M.; Knop-Gericke A.; Yigit N.; Li H.; McDermott E.; et al. Operando XAS and NAP-XPS Studies of Preferential CO Oxidation on Co_3_O_4_ and CeO_2_-Co_3_O_4_ Catalysts. J. Catal. 2016, 344, 1–15. 10.1016/j.jcat.2016.09.002.

[ref25] van Oversteeg C. H. M.; Doan H. Q.; de Groot F. M. F.; Cuk T.; Cuk T. In Situ X-Ray Absorption Spectroscopy of Transition Metal Based Water Oxidation Catalysts. Chem. Soc. Rev. 2017, 46 (1), 102–125. 10.1039/C6CS00230G. 27834973

[ref26] Terashita H.; Cezar J. C.; Ardito F. M.; Bufaiçal L. F.; Granado E. Element-Specific and Bulk Magnetism, Electronic, and Crystal Structures of La_0.70_Ca_0.30_Mn_1-x_Cr_x_O_3_. Phys. Rev. B 2012, 85 (10), 104401 10.1103/PhysRevB.85.104401.

[ref27] Toulemonde O.; Studer F.; Barnabé A.; Maignan A.; Martin C.; Raveau B. Charge States of Transition Metal in “Cr, Co and Ni” Doped Ln_0.5_Ca_0.5_MnO_3_ CMR Manganites. Eur. Phys. J. B 1998, 4 (2), 159–167. 10.1007/s100510050364.

[ref28] Al Samarai M.; Delgado-Jaime M. U.; Ishii H.; Hiraoka N.; Tsuei K.-D.; Rueff J.; Lassale-Kaiser B.; Weckhuysen B. M.; de Groot F. M. F. 1s3p Resonant Inelastic X-Ray Scattering of Cobalt Oxides and Sulfides. J. Phys. Chem. C 2016, 120 (42), 24063–24069. 10.1021/acs.jpcc.6b06444.

[ref29] Bordage A.; Trannoy V.; Proux O.; Vitoux H.; Moulin R.; Bleuzen A. In Situ Site-Selective Transition Metal K-Edge XAS: A Powerful Probe of the Transformation of Mixed-Valence Compounds. Phys. Chem. Chem. Phys. 2015, 17 (26), 17260–17265. 10.1039/C5CP02591E. 26073970

[ref30] Martin M.; Koops U.; Lakshmi N. Reactivity of Solids Studied by in Situ XAS and XRD. Solid State Ionics 2004, 172 (1), 357–363. 10.1016/j.ssi.2004.02.052.

[ref31] Martin M.; Lakshmi N.; Koops U.; Yoo H.-I. In Situ Investigations on the Oxidation of Metals. Z. fur Phys. Chem. 2007, 221 (11–12), 1499–1508. 10.1524/zpch.2007.221.11-12.1499.

[ref32] Roos B. O. The Complete Active Space SCF Method in a Fock-Matrix-Based Super-CI Formulation. Int. J. Quantum Chem. 1980, 18 (S14), 175–189. 10.1002/qua.560180822.

[ref33] Roos B. O.; Taylor P. R.; Sigbahn P. E. M. A Complete Active Space SCF Method (CASSCF) Using a Density Matrix Formulated Super-CI Approach. Chem. Phys. 1980, 48 (2), 157–173. 10.1016/0301-0104(80)80045-0.

[ref34] Siegbahn P. E. M.; Almlöf J.; Heiberg A.; Roos B. O. The Complete Active Space SCF (CASSCF) Method in a Newton–Raphson Formulation with Application to the HNO Molecule. J. Chem. Phys. 1981, 74 (4), 2384–2396. 10.1063/1.441359.

[ref35] Malmqvist P. A..; Rendell A..; Roos B. O. The Restricted Active Space Self-Consistent-Field Method, Implemented with a Split Graph Unitary Group Approach. J. Phys. Chem. 1990, 94 (14), 5477–5482. 10.1021/j100377a011.

[ref36] Angeli C.; Cimiraglia R.; Evangelisti S.; Leininger T.; Malrieu J.-P. Introduction of N-Electron Valence States for Multireference Perturbation Theory. J. Chem. Phys. 2001, 114 (23), 10252–10264. 10.1063/1.1361246.

[ref37] Sivalingam K.; Krupicka M.; Auer A. A.; Neese F. Comparison of Fully Internally and Strongly Contracted Multireference Configuration Interaction Procedures. J. Chem. Phys. 2016, 145 (5), 054104 10.1063/1.4959029. 27497536

[ref38] Malmqvist P. Å.; Pierloot K.; Shahi A. R. M.; Cramer C. J.; Gagliardi L. The Restricted Active Space Followed by Second-Order Perturbation Theory Method: Theory and Application to the Study of CuO2 and Cu2O2 Systems. J. Chem. Phys. 2008, 128 (20), 204109 10.1063/1.2920188. 18513012

[ref39] Chantzis A.; Kowalska J. K.; Maganas D.; DeBeer S.; Neese F. Ab Initio Wave Function-Based Determination of Element Specific Shifts for the Efficient Calculation of X-Ray Absorption Spectra of Main Group Elements and First Row Transition Metals. J. Chem. Theory Comput. 2018, 14 (7), 3686–3702. 10.1021/acs.jctc.8b00249. 29894196

[ref40] Maganas D.; Kowalska J. K.; Van Stappen C.; DeBeer S.; Neese F. Mechanism of L2,3-Edge X-Ray Magnetic Circular Dichroism Intensity from Quantum Chemical Calculations and Experiment—A Case Study on V(IV)/V(III) Complexes. J. Chem. Phys. 2020, 152 (11), 114107 10.1063/1.5129029. 32199419

[ref41] Van Stappen C.; Van Kuiken B. E.; Mörtel M.; Ruotsalainen K. O.; Maganas D.; Khusniyarov M. M.; DeBeer S. Correlating Valence and 2p3d RIXS Spectroscopies: A Ligand-Field Study of Spin-Crossover Iron(II). Inorg. Chem. 2024, 63 (16), 7386–7400. 10.1021/acs.inorgchem.4c00435. 38587408 PMC11040727

[ref42] Maganas D.; Kristiansen P.; Duda L.-C.; Knop-Gericke A.; DeBeer S.; Schlögl R.; Neese F. Combined Experimental and Ab Initio Multireference Configuration Interaction Study of the Resonant Inelastic X-Ray Scattering Spectrum of CO2. J. Phys. Chem. C 2014, 118 (35), 20163–20175. 10.1021/jp505628y.

[ref43] Maganas D.; Kowalska J. K.; Nooijen M.; DeBeer S.; Neese F. Comparison of Multireference Ab Initio Wavefunction Methodologies for X-Ray Absorption Edges: A Case Study on [Fe(II/III)Cl4]2–/1– Molecules. J. Chem. Phys. 2019, 150 (10), 104106 10.1063/1.5051613. 30876345

[ref44] Vidal M. L.; Pokhilko P.; Krylov A. I.; Coriani S. Equation-of-Motion Coupled-Cluster Theory to Model L-Edge X-Ray Absorption and Photoelectron Spectra. J. Phys. Chem. Lett. 2020, 11 (19), 8314–8321. 10.1021/acs.jpclett.0c02027. 32897075

[ref45] Vila F. D.; Kas J. J.; Rehr J. J.; Kowalski K.; Peng B. Equation-of-Motion Coupled-Cluster Cumulant Green’s Function for Excited States and X-Ray Spectra. Front. Chem. 2021, 9, 734945 10.3389/fchem.2021.734945. 34631660 PMC8493088

[ref46] Norman P.; Dreuw A. Simulating X-Ray Spectroscopies and Calculating Core-Excited States of Molecules. Chem. Rev. 2018, 118 (15), 7208–7248. 10.1021/acs.chemrev.8b00156. 29894157

[ref47] Helmich-Paris B. Simulating X-Ray Absorption Spectra with Complete Active Space Self-Consistent Field Linear Response Methods. Int. J. Quantum Chem. 2021, 121 (3), e26559 10.1002/qua.26559.

[ref48] Roemelt M.; Neese F. Excited States of Large Open-Shell Molecules: An Efficient, General, and Spin-Adapted Approach Based on a Restricted Open-Shell Ground State Wave Function. J. Phys. Chem. A 2013, 117 (14), 3069–3083. 10.1021/jp3126126. 23510206

[ref49] Roemelt M.; Maganas D.; DeBeer S.; Neese F. A Combined DFT and Restricted Open-Shell Configuration Interaction Method Including Spin-Orbit Coupling: Application to Transition Metal L-Edge X-Ray Absorption Spectroscopy. J. Chem. Phys. 2013, 138 (20), 204101 10.1063/1.4804607. 23742448

[ref50] Maganas D.; DeBeer S.; Neese F. Pair Natural Orbital Restricted Open-Shell Configuration Interaction (PNO-ROCIS) Approach for Calculating X-Ray Absorption Spectra of Large Chemical Systems. J. Phys. Chem. A 2018, 122 (5), 1215–1227. 10.1021/acs.jpca.7b10880. 29313679

[ref51] Maganas D.; DeBeer S.; Neese F. A Restricted Open Configuration Interaction with Singles Method To Calculate Valence-to-Core Resonant X-Ray Emission Spectra: A Case Study. Inorg. Chem. 2017, 56 (19), 11819–11836. 10.1021/acs.inorgchem.7b01810. 28920680 PMC5692824

[ref52] Kubas A.; Verkamp M.; Vura-Weis J.; Neese F.; Maganas D. Restricted Open-Shell Configuration Interaction Singles Study on M- and L-Edge X-Ray Absorption Spectroscopy of Solid Chemical Systems. J. Chem. Theory Comput. 2018, 14 (8), 4320–4334. 10.1021/acs.jctc.8b00302. 29949367

[ref53] Chilkuri V. G.; Neese F. Comparison of Many-Particle Representations for Selected-CI I: A Tree Based Approach. J. Comput. Chem. 2021, 42 (14), 982–1005. 10.1002/jcc.26518. 33764585

[ref54] Chilkuri V. G.; Neese F. Comparison of Many-Particle Representations for Selected Configuration Interaction: II. Numerical Benchmark Calculations. J. Chem. Theory Comput. 2021, 17 (5), 2868–2885. 10.1021/acs.jctc.1c00081. 33886300 PMC8279407

[ref55] Leyser da Costa Gouveia T.; Maganas D.; Neese F. Restricted Open-Shell Hartree–Fock Method for a General Configuration State Function Featuring Arbitrarily Complex Spin-Couplings. J. Phys. Chem. A 2024, 128 (25), 5041–5053. 10.1021/acs.jpca.4c00688. 38886177 PMC11215774

[ref56] Davidson E. R. The Iterative Calculation of a Few of the Lowest Eigenvalues and Corresponding Eigenvectors of Large Real-Symmetric Matrices. J. Comput. Phys. 1975, 17 (1), 87–94. 10.1016/0021-9991(75)90065-0.

[ref57] Grabenstetter J. E.; Tseng T. J.; Grein F. Generation of Genealogical Spin Eigenfunctions. Int. J. Quantum Chem. 1976, 10 (1), 143–149. 10.1002/qua.560100112.

[ref58] McLean A. D.; Liu B. Classification of Configurations and the Determination of Interacting and Noninteracting Spaces in Configuration Interaction. J. Chem. Phys. 1973, 58 (3), 1066–1078. 10.1063/1.1679288.

[ref59] Edwards W. D.; Zerner M. C. A. Generalized Restricted Open-Shell Fock Operator. Theor. Chim. Acta 1987, 72 (5), 347–361. 10.1007/BF01192227.

[ref60] Foglia N. O.; Maganas D.; Neese F. Going beyond the Electric-Dipole Approximation in the Calculation of Absorption and (Magnetic) Circular Dichroism Spectra Including Scalar Relativistic and Spin–Orbit Coupling Effects. J. Chem. Phys. 2022, 157 (8), 084120 10.1063/5.0094709. 36050038

[ref61] Neese F. The ORCA Program System. Wiley Interdiscip. Rev.: Comput. Mol. Sci. 2012, 2 (1), 73–78. 10.1002/wcms.81.

[ref62] Neese F. Software Update: The ORCA Program System, Version 4.0. Wiley Interdiscip. Rev.: Comput. Mol. Sci. 2018, 8 (1), e1327 10.1002/wcms.1327.

[ref63] Neese F.; Wennmohs F.; Becker U.; Riplinger C. The ORCA Quantum Chemistry Program Package. J. Chem. Phys. 2020, 152 (22), 224108 10.1063/5.0004608. 32534543

[ref64] Neese F. Software Update: The ORCA Program System—Version 5.0. Wiley Interdiscip. Rev.: Comput. Mol. Sci. 2022, 12 (5), e1606 10.1002/wcms.1606.

[ref65] Neese F. The SHARK Integral Generation and Digestion System. J. Comput. Chem. 2023, 44 (3), 381–396. 10.1002/jcc.26942. 35678278

[ref66] Perdew J. P. Density-Functional Approximation for the Correlation Energy of the Inhomogeneous Electron Gas. Phys. Rev. B 1986, 33 (12), 8822–8824. 10.1103/PhysRevB.33.8822. 9938299

[ref67] Becke A. D. Density-Functional Exchange-Energy Approximation with Correct Asymptotic Behavior. Phys. Rev. A 1988, 38 (6), 3098–3100. 10.1103/PhysRevA.38.3098. 9900728

[ref68] Grimme S.; Antony J.; Ehrlich S.; Krieg H. A Consistent and Accurate Ab Initio Parametrization of Density Functional Dispersion Correction (DFT-D) for the 94 Elements H-Pu. J. Chem. Phys. 2010, 132 (15), 154104 10.1063/1.3382344. 20423165

[ref69] Grimme S.; Ehrlich S.; Goerigk L. Effect of the Damping Function in Dispersion Corrected Density Functional Theory. J. Comput. Chem. 2011, 32 (7), 1456–1465. 10.1002/jcc.21759. 21370243

[ref70] Caldeweyher E.; Bannwarth C.; Grimme S. Extension of the D3 Dispersion Coefficient Model. J. Chem. Phys. 2017, 147 (3), 034112 10.1063/1.4993215. 28734285

[ref71] Caldeweyher E.; Ehlert S.; Hansen A.; Neugebauer H.; Spicher S.; Bannwarth C.; Grimme S. A Generally Applicable Atomic-Charge Dependent London Dispersion Correction. J. Chem. Phys. 2019, 150 (15), 154122 10.1063/1.5090222. 31005066

[ref72] Caldeweyher E.; Mewes J.-M.; Ehlert S.; Grimme S. Extension and Evaluation of the D4 London-Dispersion Model for Periodic Systems. Phys. Chem. Chem. Phys. 2020, 22 (16), 8499–8512. 10.1039/D0CP00502A. 32292979

[ref73] Weigend F.; Ahlrichs R. Balanced Basis Sets of Split Valence, Triple Zeta Valence and Quadruple Zeta Valence Quality for H to Rn: Design and Assessment of Accuracy. Phys. Chem. Chem. Phys. 2005, 7 (18), 3297–3305. 10.1039/b508541a. 16240044

[ref74] Neese F. An Improvement of the Resolution of the Identity Approximation for the Formation of the Coulomb Matrix. J. Comput. Chem. 2003, 24 (14), 1740–1747. 10.1002/jcc.10318. 12964192

[ref75] Chilkuri V. G.; Suaud N.; Guihéry N. High-Spin Chains and Crowns from Double-Exchange Mechanism. Crystals 2016, 6 (4), 39 10.3390/cryst6040039.

[ref76] Pipek J.; Mezey P. G. A Fast Intrinsic Localization Procedure Applicable for Ab Initio and Semiempirical Linear Combination of Atomic Orbital Wave Functions. J. Chem. Phys. 1989, 90 (9), 4916–4926. 10.1063/1.456588.

[ref77] Budiyanto E.; Yu M.; Chen M.; DeBeer S.; Rüdiger O.; Tüysüz H. Tailoring Morphology and Electronic Structure of Cobalt Iron Oxide Nanowires for Electrochemical Oxygen Evolution Reaction. ACS Appl. Energy Mater. 2020, 3 (9), 8583–8594. 10.1021/acsaem.0c01201.

