# Supplementary material for: General Spin-Restricted Open-Shell Configuration Interaction Approach: Application to Metal K-Edge X-ray Absorption Spectra of Ferro- and Antiferromagnetically Coupled Dimers
Source: J Phys Chem A. 2024 Dec 16;129(1):330–45. doi: 10.1021/acs.jpca.4c05228 (PMC11726630; doi:10.1021/acs.jpca.4c05228)
Supplement: Supplementary file 1 — jp4c05228_si_001.pdf [file jp4c05228_si_001.pdf]

Supporting Information

A General Spin Restricted Open-Shell Configuration Interaction Approach: Application to metal K-edge X-rays Absorption spectra of Ferro- and Antiferromagnetically coupled dimers

Tiago Leyser da Costa Gouveia[1], Dimitrios Maganas[1] and Frank Neese[1*]

[1] Max-Planck-Institut für Kohlenforschung, Kaiser-Wilheim-Platz 1, 45470, Mülheim an der Ruhr, Germany

[2] Max-Planck Institute for Chemical Energy Conversion, Stiftstraße 34-36, 45470, Mülheim an der Ruhr, Germany

Table of Contents

1. **Sigma Vector Matrix Elements** **S2**

2. **Study Set of Mononuclear Molecules** **S5**

3. **Calculated pre-edge of test set** **S6**

4. **Representative Inputs** **S7**

   A. **GS-ROCIS HS** **S7**

   B. **GS-ROCIS AF** **S8**

5. **Molecular Geometries of Dimers** **S9**

   A. **$[Ni^{II}(O)(H2O)_5]_n^{2+}$** **S9**

      i. n=1 Ni: S9
      ii. n=2 Ni: S9
      iii. n=3 Ni: S9
      iv. n=4 Ni: S10
      v. n=5 Ni: S11
      vi. n=6 Ni: S12
      vii. n=7 Ni: S13
      viii. n=8 Ni: S14
      ix. n=9 Ni: S15
      x. n=10 Ni: S16

6. **Molecular Geometry of $[Cu_2(\mu\text{-F})(H_2O)_6]^{3+}$** **S17**

7. **Molecular Geometry of $[Ni2(\mu\text{-O})_2(H_2O)_8]$** **S17**

8. **Molecular Geometry of the Co_3O_4 quantum cluster** **S18**

1. Sigma Vector Matrix Elements

$$\langle \boldsymbol{\Phi_0}|\boldsymbol{H}|\boldsymbol{\Phi_0}\rangle = \sum_i^{DOMOs} 2F_{ii}^C + \sum_t^{SOMOs} F_{tt}^O - \sum_i^{DOMOs}\sum_j^{DOMOs}[2(ii|jj)-(ij|ij)] - \sum_i^{DOMOs}\sum_t^{SOMOs}[2(ii|tt)-(it|it)]$$

$$-\frac{1}{2}\sum_t^{SOMOs}\sum_u^{SOMOs}[(tt|uu)-(tu|tu)] + \frac{1}{2}\sum_t^{SOMOs}\sum_{u\neq t}^{SOMOs}(tu|tu)\langle\Phi_0|E_u^t E_t^u|\Phi_0\rangle$$

$$\langle \boldsymbol{\Phi_0}|\boldsymbol{H}|\boldsymbol{\Phi_i^t}\rangle = [F_{ti}^O + (ti|tt)]\langle\Phi_0|E_i^t|\Phi_i^t\rangle + \sum_{u\neq t}^{SOMOs}(iu|tu)\langle\Phi_0|E_i^u E_u^t|\Phi_i^t\rangle$$

$$\langle \boldsymbol{\Phi_0}|\boldsymbol{H}|\boldsymbol{\Phi_t^a}\rangle = F_{at}^O\langle\Phi_0|E_t^a|\Phi_t^a\rangle + \sum_{u\neq t}^{SOMOs}(tu|au)\langle\Phi_0|E_t^u E_u^a|\Phi_t^a\rangle$$

$$\langle \boldsymbol{\Phi_0}|\boldsymbol{H}|\boldsymbol{\Phi_i^a}\rangle = F_{ai}^O\langle\Phi_0|E_i^a|\Phi_i^a\rangle + \sum_t^{SOMOs}(it|at)\langle\Phi_0|E_i^t E_t^a|\Phi_i^a\rangle$$

$$\langle \boldsymbol{\Phi_0}|\boldsymbol{H}|\boldsymbol{\Phi_{ui}^{at}}\rangle = (au|ti)\langle\Phi_0|E_u^a E_i^t|\Phi_{ui}^{at}\rangle + (ai|tu)\langle\Phi_0|E_i^a E_u^t|\Phi_{ui}^{at}\rangle$$

$$\langle \boldsymbol{\Phi_i^t}|\boldsymbol{H}|\boldsymbol{\Phi_i^t}\rangle - \boldsymbol{E_0} = F_{tt}^I - F_{ii}^I - (ii|tt) + (it|it) + \sum_{u\neq t}^{SOMOs}(iu|iu)\langle\Phi_i^t|E_i^u E_u^i|\Phi_i^t\rangle$$

$$+\frac{1}{2}\sum_{u\neq t}^{SOMOs}\sum_{v\neq t,u}^{SOMOs}(uv|uv)\langle\Phi_i^t|E_v^u E_u^v|\Phi_i^t\rangle - \frac{1}{2}\sum_t^{SOMOs}\sum_{u\neq t}^{SOMOs}(tu|tu)\langle\Phi_0|E_u^t E_t^u|\Phi_0\rangle$$

$$\langle \boldsymbol{\Phi_i^t}|\boldsymbol{H}|\boldsymbol{\Phi_j^t}\rangle = [F_{ij}^O + (ij|tt)]\langle\Phi_i^t|E_j^i|\Phi_j^t\rangle + \sum_{u\neq t}^{SOMOs}(ju|iu)\langle\Phi_i^t|E_j^u E_u^i|\Phi_j^t\rangle$$

$$\langle \boldsymbol{\Phi_i^t}|\boldsymbol{H}|\boldsymbol{\Phi_i^u}\rangle = [F_{ut}^O + (ut|tt) + (ut|uu) - (ut|ii)]\langle\Phi_i^t|E_t^u|\Phi_i^u\rangle + (ti|ui)\langle\Phi_i^t|E_t^i E_i^u|\Phi_i^u\rangle$$

$$+\sum_{v\neq t,u}^{SOMOs}(tv|uv)\langle\Phi_i^t|E_t^v E_v^u|\Phi_i^u\rangle$$

$$\langle \boldsymbol{\Phi_i^t}|\boldsymbol{H}|\boldsymbol{\Phi_j^u}\rangle = (it|uj)\langle\Phi_i^t|E_t^i E_j^u|\Phi_j^u\rangle + (ij|ut)\langle\Phi_i^t|E_j^i E_t^u|\Phi_j^u\rangle$$

$$\langle \boldsymbol{\Phi_i^t}|\boldsymbol{H}|\boldsymbol{\Phi_t^a}\rangle = (at|it)\langle\Phi_i^t|E_t^a E_t^i|\Phi_t^a\rangle$$

$$\langle \boldsymbol{\Phi_i^t}|\boldsymbol{H}|\boldsymbol{\Phi_u^a}\rangle = (it|au)\langle\Phi_i^t|E_t^i E_u^a|\Phi_u^a\rangle + (iu|at)\langle\Phi_i^t|E_u^i E_t^a|\Phi_u^a\rangle$$

$$\langle \boldsymbol{\Phi_i^t}|\boldsymbol{H}|\boldsymbol{\Phi_i^a}\rangle = \left[F_{at}^I + (at|ii) - \sum_{u\neq t}^{SOMOs}(tu|au)\right]\langle\Phi_i^t|E_t^a|\Phi_i^a\rangle + (ti|ai)\langle\Phi_i^t|E_t^i E_i^a|\Phi_i^a\rangle$$

$$+\sum_{u\neq t}^{SOMOs}(tu|au)\langle\Phi_i^t|E_t^u E_u^a|\Phi_i^a\rangle$$

$$\langle \boldsymbol{\Phi_i^t}|\boldsymbol{H}|\boldsymbol{\Phi_j^a}\rangle = (it|aj)\langle\Phi_i^t|E_t^i E_j^a|\Phi_j^a\rangle + (ij|at)\langle\Phi_i^t|E_j^i E_t^a|\Phi_j^a\rangle$$

$$\langle \boldsymbol{\Phi_i^t}|\boldsymbol{H}|\boldsymbol{\Phi_{ui}^{at}}\rangle = [F_{au}^O - (au|ii) + (au|tt)]\langle\Phi_i^t|E_u^a|\Phi_{ui}^{at}\rangle + (ui|ai)\langle\Phi_i^t|E_u^i E_i^a|\Phi_{ui}^{at}\rangle$$

$$+\sum_{v\neq t,u}^{SOMOs}(uv|av)\langle\Phi_i^t|E_u^v E_v^a|\Phi_{ui}^{at}\rangle$$

$$\langle \boldsymbol{\Phi_i^t}|\boldsymbol{H}|\boldsymbol{\Phi_{ti}^{au}}\rangle = (at|ut)\langle\Phi_i^t|E_t^a E_t^u|\Phi_{ti}^{au}\rangle$$

$$\langle \boldsymbol{\Phi_i^t}|\boldsymbol{H}|\boldsymbol{\Phi_{uj}^{at}}\rangle = (ij|au)\langle\Phi_i^t|E_j^i E_u^a|\Phi_{uj}^{at}\rangle + (iu|aj)\langle\Phi_i^t|E_u^i E_j^a|\Phi_{uj}^{at}\rangle$$

$$\langle \boldsymbol{\Phi_i^t}|\boldsymbol{H}|\boldsymbol{\Phi_{vi}^{au}}\rangle = (av|ut)\langle\Phi_i^t|E_v^a E_t^u|\Phi_{vi}^{au}\rangle + (at|uv)\langle\Phi_i^t|E_t^a E_v^u|\Phi_{vi}^{au}\rangle$$

$$\langle \boldsymbol{\Phi_t^a}|\boldsymbol{H}|\boldsymbol{\Phi_t^a}\rangle - \boldsymbol{E_0} = F_{aa}^O - F_{tt}^O - (tt|aa) + (ta|ta) + \sum_{u\neq t}^{SOMOs}(au|au)\langle\Phi_t^a|E_a^u E_u^a|\Phi_t^a\rangle + \frac{1}{2}\sum_{u\neq t}^{SOMOs}\sum_{v\neq t,u}^{SOMOs}(uv|uv)\langle\Phi_t^a|E_v^u E_u^v|\Phi_t^a\rangle - \frac{1}{2}\sum_{t}^{SOMOs}\sum_{u\neq t}^{SOMOs}(tu|tu)\langle\Phi_0|E_u^t E_t^u|\Phi_0\rangle$$

$$\langle \boldsymbol{\Phi_t^a}|\boldsymbol{H}|\boldsymbol{\Phi_t^b}\rangle = [F_{ba}^O - (ba|tt) + (bt|at)]\langle\Phi_t^a|E_a^b|\Phi_t^b\rangle + \sum_{u\neq t}^{SOMOs}(au|bu)\langle\Phi_t^a|E_a^u E_u^b|\Phi_t^b\rangle$$

$$\langle \boldsymbol{\Phi_t^a}|\boldsymbol{H}|\boldsymbol{\Phi_u^a}\rangle = [F_{tu}^O - (tu|aa) + (ta|ua)]\langle\Phi_t^a|E_u^t|\Phi_u^a\rangle + (ua|ta)\langle\Phi_t^a|E_u^a E_a^t|\Phi_u^a\rangle + \sum_{v\neq t,u}^{SOMOs}(uv|tv)\langle\Phi_t^a|E_u^v E_v^t|\Phi_u^a\rangle$$

$$\langle \boldsymbol{\Phi_t^a}|\boldsymbol{H}|\boldsymbol{\Phi_u^b}\rangle = (ta|bu)\langle\Phi_t^a|E_a^t E_u^b|\Phi_u^b\rangle + (tu|ba)\langle\Phi_t^a|E_u^t E_a^b|\Phi_u^b\rangle$$

$$\langle \boldsymbol{\Phi_t^a}|\boldsymbol{H}|\boldsymbol{\Phi_i^a}\rangle = [F_{ti}^O + (ti|aa) - (ta|ia)]\langle\Phi_t^a|E_i^t|\Phi_i^a\rangle + (ta|ia)\langle\Phi_t^a|E_i^a E_a^t|\Phi_i^a\rangle + \sum_{u\neq t}^{SOMOs}(iu|tu)\langle\Phi_t^a|E_i^u E_u^t|\Phi_i^a\rangle$$

$$\langle \boldsymbol{\Phi_t^a}|\boldsymbol{H}|\boldsymbol{\Phi_i^b}\rangle = (ta|bi)\langle\Phi_t^a|E_a^t E_i^b|\Phi_i^b\rangle + (ti|ba)\langle\Phi_t^a|E_i^t E_a^b|\Phi_i^b\rangle$$

$$\langle \boldsymbol{\Phi_t^a}|\boldsymbol{H}|\boldsymbol{\Phi_{ui}^{at}}\rangle = (ti|tu)\langle\Phi_t^a|E_i^t E_u^t|\Phi_{ui}^{at}\rangle$$

$$\langle \boldsymbol{\Phi_t^a}|\boldsymbol{H}|\boldsymbol{\Phi_{ti}^{au}}\rangle = [F_{ui}^O + (ui|uu) + (ui|aa) - (ua|ia)]\langle\Phi_t^a|E_i^u|\Phi_{ti}^{au}\rangle + (ua|ia)\langle\Phi_t^a|E_i^a E_a^u|\Phi_{ti}^{au}\rangle + \sum_{v\neq t,u}^{SOMOs}(iv|uv)\langle\Phi_t^a|E_i^v E_v^u|\Phi_{ti}^{au}\rangle$$

$$\langle \boldsymbol{\Phi_t^a}|\boldsymbol{H}|\boldsymbol{\Phi_{ti}^{bu}}\rangle = (ui|ba)\langle\Phi_t^a|E_i^u E_a^b|\Phi_{ti}^{bu}\rangle + (ua|bi)\langle\Phi_t^a|E_a^u E_i^b|\Phi_{ti}^{bu}\rangle$$

$$\langle \boldsymbol{\Phi_t^a}|\boldsymbol{H}|\boldsymbol{\Phi_{vi}^{au}}\rangle = (ti|uv)\langle\Phi_t^a|E_i^t E_v^u|\Phi_{vi}^{au}\rangle + (tv|ui)\langle\Phi_t^a|E_v^t E_i^u|\Phi_{vi}^{au}\rangle$$

$$\langle \boldsymbol{\Phi_i^a}|\boldsymbol{H}|\boldsymbol{\Phi_i^a}\rangle - \boldsymbol{E_0} = F_{aa}^O - F_{ii}^I - (ii|aa) + (ia|ia)\langle\Phi_i^a|E_i^a E_a^i|\Phi_i^a\rangle + \sum_{t}^{SOMOs}(it|it)\langle\Phi_i^a|E_t^i E_i^t|\Phi_i^a\rangle + \sum_{t}^{SOMOs}(at|at)\langle\Phi_i^a|E_t^a E_a^t|\Phi_i^a\rangle + \frac{1}{2}\sum_{t}^{SOMOs}\sum_{u\neq t}^{SOMOs}(tu|tu)\langle\Phi_i^a|E_u^t E_t^u|\Phi_i^a\rangle - \frac{1}{2}\sum_{t}^{SOMOs}\sum_{u\neq t}^{SOMOs}(tu|tu)\langle\Phi_0|E_u^t E_t^u|\Phi_0\rangle$$

$$\langle \boldsymbol{\Phi_i^a}|\boldsymbol{H}|\boldsymbol{\Phi_j^a}\rangle = [F_{ij}^O + (ij|aa) - (ia|ja)]\langle\Phi_i^a|E_j^i|\Phi_j^a\rangle + (ia|ja)\langle\Phi_i^a|E_a^i E_j^a|\Phi_j^a\rangle + \sum_{t}^{SOMOs}(it|jt)\langle\Phi_i^a|E_t^i E_j^t|\Phi_j^a\rangle$$

$$\langle \boldsymbol{\Phi_i^a}|\boldsymbol{H}|\boldsymbol{\Phi_i^b}\rangle = [F_{ba}^O - (ba|ii)]\langle\Phi_i^a|E_a^b|\Phi_i^b\rangle + (bi|ai)\langle\Phi_i^a|E_i^b E_a^i|\Phi_i^b\rangle + \sum_{t}^{SOMOs}(bt|at)\langle\Phi_i^a|E_i^b E_a^i|\Phi_i^b\rangle$$

$$\langle \boldsymbol{\Phi_i^a}|\boldsymbol{H}|\boldsymbol{\Phi_j^b}\rangle = (ia|bj)\langle\Phi_i^a|E_a^i E_j^b|\Phi_j^b\rangle + (ij|ba)\langle\Phi_i^a|E_j^i E_a^b|\Phi_j^b\rangle$$

$$\langle \boldsymbol{\Phi_i^a}|\boldsymbol{H}|\boldsymbol{\Phi_{ui}^{at}}\rangle = [F_{tu}^O - (tu|ii) + (tu|tt) + (tu|aa) - (ta|ua)]\langle\Phi_i^a|E_u^t|\Phi_{ui}^{at}\rangle + (ui|ti)\langle\Phi_i^a|E_u^i E_i^t|\Phi_{ui}^{at}\rangle + (ua|ta)\langle\Phi_i^a|E_u^a E_a^t|\Phi_{ui}^{at}\rangle + \sum_{v\neq t,u}^{SOMOs}(uv|tv)\langle\Phi_i^a|E_v^t E_u^v|\Phi_{ui}^{at}\rangle$$

$$\langle \boldsymbol{\Phi_i^a}|\boldsymbol{H}|\boldsymbol{\Phi_{uj}^{at}}\rangle = (iu|tj)\langle\Phi_i^a|E_u^i E_j^t|\Phi_{uj}^{at}\rangle + (ij|tu)\langle\Phi_i^a|E_j^i E_u^t|\Phi_{uj}^{at}\rangle$$

$$\langle \boldsymbol{\Phi}_i^a|\boldsymbol{H}|\boldsymbol{\Phi}_{ui}^{bt}\rangle = (ta|bu)\langle \Phi_i^a|E_a^t E_u^b|\Phi_{ui}^{bt}\rangle + (tu|ba)\langle \Phi_i^a|E_u^t E_a^b|\Phi_{ui}^{bt}\rangle$$

$$\langle \boldsymbol{\Phi}_{ui}^{at}|\boldsymbol{H}|\boldsymbol{\Phi}_{ui}^{at}\rangle - \boldsymbol{E_0} = F_{aa}^O - F_{ii}^I + F_{tt}^I - F_{uu}^O - (ii|tt) + (it|it) - (tt|uu) + (ii|uu) - (ii|aa)$$

$$+ (tt|aa) - (uu|aa) + (ua|ua) + (ia|ia)\langle \Phi_{ui}^{at}|E_a^i E_i^a|\Phi_{ui}^{at}\rangle + \sum_{v\neq t,u}^{SOMOs}(iv|iv)\langle \Phi_{ui}^{at}|E_i^v E_v^i|\Phi_{ui}^{at}\rangle$$

$$+ \sum_{v\neq t,u}^{SOMOs}(av|av)\langle \Phi_{ui}^{at}|E_a^v E_v^a|\Phi_{ui}^{at}\rangle + \frac{1}{2}\sum_{v\neq t,u}^{SOMOs}\sum_{w\neq t,u,v}^{SOMOs}(vw|vw)\langle \Phi_{ui}^{at}|E_w^v E_v^w|\Phi_{ui}^{at}\rangle$$

$$-\frac{1}{2}\sum_{t}^{SOMOs}\sum_{u\neq t}^{SOMOs}(tu|tu)\langle \Phi_0|E_u^t E_t^u|\Phi_0\rangle$$

$$\langle \boldsymbol{\Phi}_{ui}^{at}|\boldsymbol{H}|\boldsymbol{\Phi}_{uj}^{at}\rangle = \left[F_{ij}^O + (ij|tt) - (ij|uu) + (iu|ju) + (ij|aa) - (ia|ja)\right]\langle \Phi_{ui}^{at}|E_j^i|\Phi_{uj}^{at}\rangle$$

$$+ (ia|ja)\langle \Phi_{ui}^{at}|E_j^a E_a^i|\Phi_{uj}^{at}\rangle + \sum_{v\neq t,u}^{SOMOs}(iv|jv)\langle \Phi_{ui}^{at}|E_v^i E_j^v|\Phi_{uj}^{at}\rangle$$

$$\langle \boldsymbol{\Phi}_{ui}^{at}|\boldsymbol{H}|\boldsymbol{\Phi}_{ui}^{bt}\rangle = [F_{ba}^O - (ba|ii) + (ba|tt) - (ba|uu) + (bu|au)]\langle \Phi_{ui}^{at}|E_a^b|\Phi_{ui}^{bt}\rangle + (bi|ai)\langle \Phi_{ui}^{at}|E_a^i E_i^b|\Phi_{ui}^{bt}\rangle$$

$$+ \sum_{v\neq t,u}^{SOMOs}(bv|av)\langle \Phi_{ui}^{at}|E_v^b E_a^v|\Phi_{ui}^{bt}\rangle$$

$$\langle \boldsymbol{\Phi}_{ui}^{at}|\boldsymbol{H}|\boldsymbol{\Phi}_{ui}^{av}\rangle = [F_{vt}^O - (vt|ii) + (vt|tt) + (vt|vv) - (vt|uu) + (vt|aa)]\langle \Phi_{ui}^{at}|E_t^v|\Phi_{ui}^{av}\rangle$$

$$+ (vi|ti)\langle \Phi_{ui}^{at}|E_t^i E_i^v|\Phi_{ui}^{av}\rangle + (va|ta)\langle \Phi_{ui}^{at}|E_t^a E_a^v|\Phi_{ui}^{av}\rangle + \sum_{w\neq t,u,v}^{SOMOs}(vw|tw)\langle \Phi_{ui}^{at}|E_t^w E_w^v|\Phi_{ui}^{av}\rangle$$

$$\langle \boldsymbol{\Phi}_{ui}^{at}|\boldsymbol{H}|\boldsymbol{\Phi}_{vi}^{at}\rangle = [F_{uv}^O - (uv|ii) + (uv|tt) + (uv|aa) - (ua|va)]\langle \Phi_{ui}^{at}|E_v^u|\Phi_{vi}^{at}\rangle + (vi|ui)\langle \Phi_{ui}^{at}|E_v^i E_i^u|\Phi_{vi}^{at}\rangle$$

$$+ (va|ua)\langle \Phi_{ui}^{at}|E_v^a E_a^u|\Phi_{vi}^{at}\rangle + \sum_{w\neq t,u,v}^{SOMOs}(vw|uw)\langle \Phi_{ui}^{at}|E_v^w E_w^u|\Phi_{vi}^{at}\rangle$$

$$\langle \boldsymbol{\Phi}_{ui}^{at}|\boldsymbol{H}|\boldsymbol{\Phi}_{ti}^{au}\rangle = \frac{1}{2}(ut|ut)\langle \Phi_{ui}^{at}|E_t^u E_t^u|\Phi_{ti}^{au}\rangle$$

$$\langle \boldsymbol{\Phi}_{ui}^{at}|\boldsymbol{H}|\boldsymbol{\Phi}_{vi}^{au}\rangle = (ut|uv)\langle \Phi_{ui}^{at}|E_v^u E_t^u|\Phi_{vi}^{au}\rangle$$

$$\langle \boldsymbol{\Phi}_{ui}^{at}|\boldsymbol{H}|\boldsymbol{\Phi}_{ti}^{av}\rangle = (ut|vt)\langle \Phi_{ui}^{at}|E_t^v E_t^u|\Phi_{ti}^{av}\rangle$$

$$\langle \boldsymbol{\Phi}_{ui}^{at}|\boldsymbol{H}|\boldsymbol{\Phi}_{wi}^{av}\rangle = (ut|vw)\langle \Phi_{ui}^{at}|E_t^u E_w^v|\Phi_{wi}^{av}\rangle + (uw|vt)\langle \Phi_{ui}^{at}|E_w^u E_t^v|\Phi_{wi}^{av}\rangle$$

$$\langle \boldsymbol{\Phi}_{ui}^{at}|\boldsymbol{H}|\boldsymbol{\Phi}_{uj}^{bt}\rangle = (ij|ba)\langle \Phi_{ui}^{at}|E_j^i E_a^b|\Phi_{uj}^{bt}\rangle + (ia|bj)\langle \Phi_{ui}^{at}|E_a^i E_j^b|\Phi_{uj}^{bt}\rangle$$

$$\langle \boldsymbol{\Phi}_{ui}^{at}|\boldsymbol{H}|\boldsymbol{\Phi}_{uj}^{bv}\rangle = (ij|vt)\langle \Phi_{ui}^{at}|E_j^i E_t^v|\Phi_{uj}^{bv}\rangle + (it|vj)\langle \Phi_{ui}^{at}|E_t^i E_j^v|\Phi_{uj}^{bv}\rangle$$

$$\langle \boldsymbol{\Phi}_{ui}^{at}|\boldsymbol{H}|\boldsymbol{\Phi}_{vj}^{at}\rangle = (ij|uv)\langle \Phi_{ui}^{at}|E_j^i E_v^u|\Phi_{vj}^{at}\rangle + (iv|uj)\langle \Phi_{ui}^{at}|E_v^i E_j^u|\Phi_{vj}^{at}\rangle$$

$$\langle \boldsymbol{\Phi}_{ui}^{at}|\boldsymbol{H}|\boldsymbol{\Phi}_{ui}^{bv}\rangle = (vt|ba)\langle \Phi_{ui}^{at}|E_t^v E_a^b|\Phi_{ui}^{bv}\rangle + (va|bt)\langle \Phi_{ui}^{at}|E_a^v E_t^b|\Phi_{ui}^{bv}\rangle$$

$$\langle \boldsymbol{\Phi}_{ui}^{at}|\boldsymbol{H}|\boldsymbol{\Phi}_{vi}^{bt}\rangle = (uv|ba)\langle \Phi_{ui}^{at}|E_v^u E_a^b|\Phi_{iv}^{bt}\rangle + (ua|bv)\langle \Phi_{ui}^{at}|E_a^u E_v^b|\Phi_{vi}^{bt}\rangle$$

2. Study Set of Mononuclear Molecules

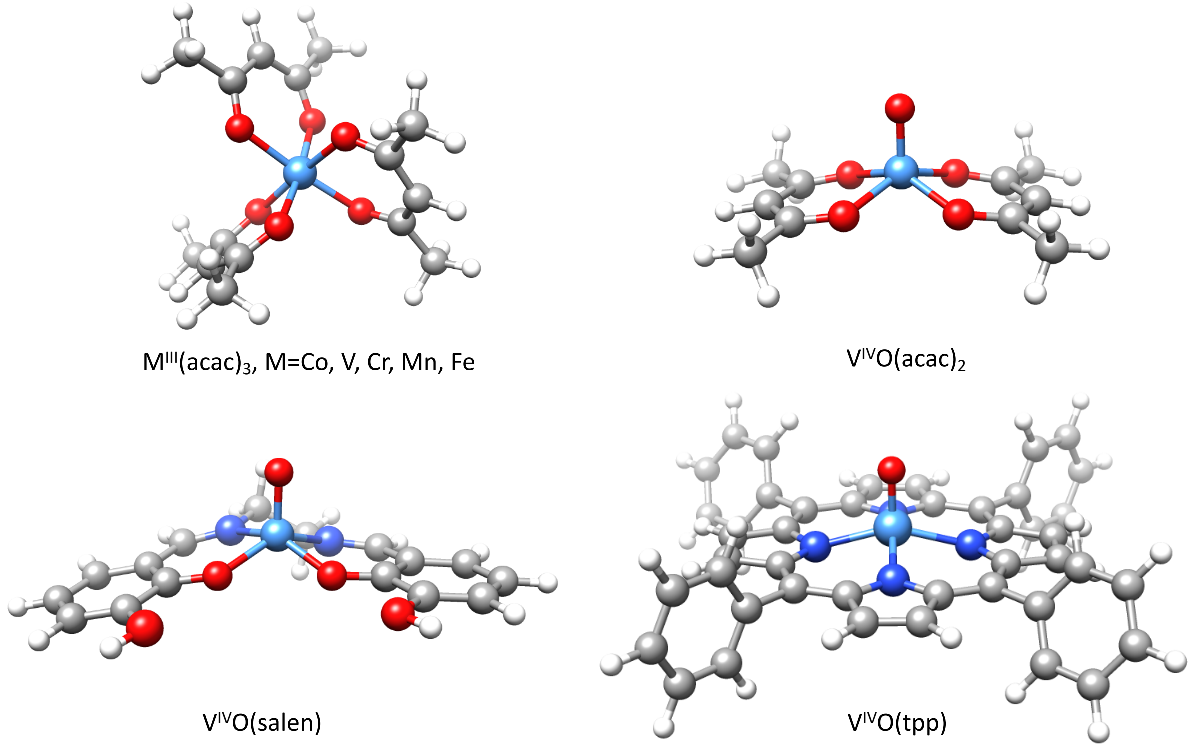

Figure S1: Study set of mononuclear molecules consisting subset A. Namely the S=0 $Co^{(III)}(acac)_3$, the S=1 $V^{(IV)}O(acac)_2$, $V^{(IV)}O$(salen) and $V^{(IV)}O$(TPP), the S=2 $V^{(III)}(acac)_3$, the S=3 $Cr^{(III)}(acac)_3$, the S=4 $Mn^{(III)}(acac)_3$ and the S=5 $Fe^{(III)}(acac)_3$. Element color coding H (white), C (gray), N (blue), O (red), M=Co, V, Cr, Mn, Fe (cyan).

3. Calculated pre-edge of test set

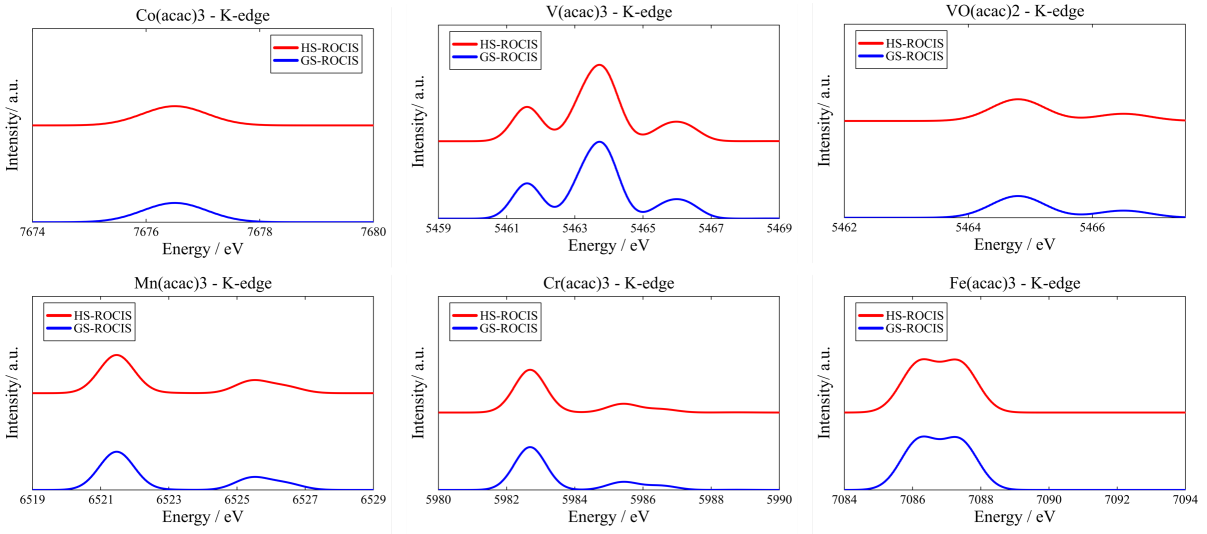

Figure S2: HS-ROCIS and GS-ROCIS calculated pre-edge spectra of the mononuclear test set of molecules.

4. Representative Inputs

A. GS-ROCIS HS

```
# Ni chain 2 High Spin GenROCIS

! def2-SVP def2-SVP/C TightSCF

%pal
 nproc 8
end

%scf
 HFTyp ROHF
 ROHF_CASE HIGHSPIN
 ROHF_NEL[1] 4
 ROHF_NORB[1] 4
 MaxIter 500
End

%rocis
 #---------------
 # GSROCIS Flags
 #---------------
 DoGenROCIS true
 ReferenceMult 5
 #---------------
 # PNO Flags
 #---------------
 #DoPNO true
 #XASElems 0
 #TCutPNO 1e-11
 #---------------
 # CI Flags
 #---------------
 NRoots 100
 Orbwin 0,0,0,2000
 #---------------
 # General Flags
 #---------------
 Maxcore 8192
 DoRI true
 #---------------
 # Spectroscopy Flags
 #---------------
 DoHigherMoments true
 DoDipoleVelocity true
 DoFullSemiClassical true

end


* xyzfile 2 5 ni2.xyz
```

B. GS-ROCIS AF

```
# Ni chain 2 Antiferromagnetic GenROCIS

! def2-SVP def2-SVP/C TightSCF

%pal
 nproc 8
end

%scf
 HFTyp ROHF
 ROHF_CASE AF_CSF
 ROHF_AFORBS 2,2
 MaxIter 500
End

%rocis
 #--------------
 # GSROCIS Flags
 #--------------
 DoGenROCIS true
 ReferenceMult 1
 #--------------
 # PNO Flags
 #--------------
 #DoPNO true
 #XASElems 0
 #TCutPNO 1e-11
 #--------------
 # CI Flags
 #--------------
 NRoots 100
 Orbwin 0,0,0,2000
 #--------------
 # General Flags
 #--------------
 Maxcore 8192
 DoRI true
 #--------------
 # Spectroscopy Flags
 #--------------
 DoHigherMoments true
 DoDipoleVelocity true
 DoFullSemiClassical true

End


* xyzfile 2 5 ni2.xyz
```

5. Molecular Geometries of Dimers

A. $[Ni^{II}(O)(H2O)_5]_n^{2+}$

i. n=1 Ni:

```
Ni  0.00000000000000     0.00000000000000     0.00000000000000
O   2.08738999752000     0.00000000000000     0.00000000000000
O  −2.08738999752000     0.00000004408251     0.00000000000000
O  −0.00000000145727     2.08738999752000     0.00000000000000
O   0.00000000072864    −2.08738999752000    −0.00000000000000
O  −0.00000000072864     0.00000000072864    −2.08738999752000
O   0.00000000072864     0.00000000072864     2.08738999752000
H   2.65763473155719     0.78999235683467    −0.00000131854335
H   2.65763485984455    −0.78999226522986    −0.00000000000000
H   0.00000000076434     2.65763446560963    −0.78999254420468
H   0.00000081701211     2.65763458772359     0.78999243939734
H  −0.78999215339847     0.00000066219845     2.65763502439174
H   0.78999239892974     0.00000000092769     2.65763469199836
H  −2.65763480169159     0.78999234525531     0.00000098542597
H  −2.65763477981872    −0.78999227327541    −0.00000000000000
H  −0.00000015005059    −2.65763446565846     0.78999254456285
H  −0.00000085447791    −2.65763459603914    −0.78999243697144
H  −0.78999241156412    −0.00000009255474    −2.65763467469341
H   0.78999214302993     0.00000000092769    −2.65763503766959
```

ii. n=2 Ni:

```
Ni  0.00023500000000    −0.03373900000000     0.01222700000000
O   2.08762500000000    −0.03373900000000     0.01238000000000
O  −2.08715500000000    −0.03373900000000     0.01207400000000
O   0.00023400000000     2.05353900000000     0.03382800000000
O   0.00023700000000    −2.12101700000000    −0.00937400000000
O   0.00038800000000    −0.01213900000000    −2.07505100000000
O   0.00008300000000    −0.05534000000000     2.09950500000000
H   2.61058100000000     0.71468400000000    −0.32528900000000
H   2.56534500000000    −0.85014500000000    −0.21914900000000
H  −0.00163000000000     2.88884100000000    −0.46497400000000
H  −0.00089600000000     2.23334200000000     0.99682200000000
H  −2.61018800000000     0.71460900000000    −0.32563500000000
H  −2.56494200000000    −0.85019200000000    −0.21913500000000
H  −0.00038700000000    −2.40844200000000     0.92301900000000
H  −0.00104300000000    −2.88919300000000    −0.60427300000000
H  −0.77788800000000     0.16732800000000    −2.63125000000000
H   0.77877800000000     0.16729300000000    −2.63110300000000
Ni −0.00007000000000    −0.07694100000000     4.18678400000000
O  −0.00007100000000     2.01033700000000     4.20838500000000
O  −0.00006900000000    −2.16421900000000     4.16518300000000
O  −0.00022200000000    −0.09854200000000     6.27406200000000
O   2.08732000000000    −0.07694100000000     4.18693600000000
O  −2.08746000000000    −0.07694100000000     4.18663100000000
H   0.77786000000000     2.56448000000000     4.39857400000000
H  −0.77701800000000     2.56455500000000     4.40237500000000
H  −0.78503000000000    −2.62371100000000     4.51544300000000
H   0.78502100000000    −2.62366600000000     4.51521700000000
H   0.00089400000000     0.65369200000000     6.89081700000000
H  −0.00039400000000    −0.90243900000000     6.82099200000000
H   2.69821900000000     0.57470400000000     4.57292600000000
H   2.09496900000000     0.02452600000000     3.20133300000000
H  −2.69838200000000     0.57470800000000     4.57257500000000
H  −2.09494600000000     0.02444600000000     3.20101500000000
```

iii. n=3 Ni:

```
Ni −0.02982700000000    −0.01276000000000    −0.00029800000000
O   2.05735600000000    −0.01482900000000    −0.02964200000000
O  −2.11701000000000    −0.01069000000000     0.02904700000000
O  −0.02764500000000     2.07461400000000     0.00772500000000
O  −0.03200900000000    −2.10013300000000    −0.00832000000000
O  −0.05916300000000    −0.00470700000000    −2.08746600000000
O  −0.00049100000000    −0.02081200000000     2.08687100000000
H   2.58156100000000     0.76700300000000    −0.28339700000000
H   1.95713400000000     0.01326100000000     0.96063300000000
H   0.42762100000000     2.65403800000000    −0.63063500000000
H   0.32787200000000     2.24275300000000     0.90379900000000
H  −2.45395000000000     0.89258600000000    −0.12579300000000
H  −2.05016500000000    −0.10005400000000     1.01314300000000
H  −0.02157400000000    −2.12294300000000     0.97976200000000
H   0.81299100000000    −2.50513000000000    −0.28193300000000
H  −0.85823200000000    −0.42713300000000    −2.45500200000000
H   0.69116200000000    −0.40004200000000    −2.56706100000000
Ni  0.02884500000000    −0.02886500000000     4.17403900000000
O   0.03102800000000     2.05850900000000     4.18206200000000
O   0.02666300000000    −2.11623800000000     4.16601600000000
O   0.05818200000000    −0.03691800000000     6.26120700000000
O   2.11602800000000    −0.03093400000000     4.14469500000000
O  −2.05833700000000    −0.02679500000000     4.20338300000000
H  −0.68230600000000     2.53857200000000     4.63874800000000
H  −0.19549700000000     2.02716300000000     3.21930200000000
H  −0.89097700000000    −2.39596700000000     3.98828400000000
H   0.11777600000000    −2.09627500000000     5.14794900000000
H   2.40054300000000     0.88319500000000     4.33265500000000
H   2.07654200000000    −0.10517600000000     3.16187700000000
H  −2.00636500000000     0.17844500000000     5.17019600000000
H  −2.54573200000000     0.69777300000000     3.77292500000000
Ni  0.08751800000000    −0.04497000000000     8.34837600000000
O   0.08970000000000     2.04240300000000     8.35639800000000
O   0.08533500000000    −2.13234400000000     8.34035300000000
O  −1.99966500000000    −0.04290100000000     8.37772000000000
O   2.17470000000000    −0.04704000000000     8.31903200000000
O   0.11685300000000    −0.05302300000000    10.43554400000000
H   0.55164200000000     0.73567800000000    10.81096300000000
H   0.51387900000000    −0.81514800000000    10.89472800000000
H  −0.80858600000000     2.38131100000000     8.53407700000000
H   0.16097700000000     1.99316500000000     7.36982800000000
H  −0.69708400000000    −2.65468000000000     8.59617100000000
H   0.04249700000000    −2.01304500000000     7.35259000000000
H  −2.19089500000000    −0.38382700000000     7.48086900000000
H  −2.57156900000000    −0.49953600000000     9.02187800000000
H   2.58306100000000    −0.89721200000000     8.57071000000000
H   2.17961100000000    −0.04034000000000     7.33064900000000
```

iv. n=4 Ni:

```
Ni  0.03694200000000      0.09357200000000      0.00222500000000
O   2.12135500000000     −0.01784400000000      0.00491200000000
O  −2.04747100000000      0.20498800000000     −0.00046200000000
O   0.14831400000000      2.17781900000000      0.02883600000000
O  −0.07443100000000     −1.99067500000000     −0.02438600000000
O   0.04104500000000      0.12000200000000     −2.08499300000000
O   0.03283800000000      0.06714200000000      2.08944400000000
H   2.06613900000000      0.02448100000000      0.99628500000000
H   2.59947300000000     −0.83679000000000     −0.21611600000000
H  −0.71274800000000      2.60022400000000     −0.15529600000000
H   0.18084200000000      2.07133100000000      1.01667500000000
H  −1.93148200000000      0.20515300000000      0.99253600000000
H  −2.45300400000000     −0.65762100000000     −0.21171400000000
H   0.13403100000000     −2.01507800000000      0.94107000000000
H   0.56291700000000     −2.55589600000000     −0.49637600000000
H  −0.79411700000000      0.33133800000000     −2.53968200000000
H   0.70572600000000      0.73335500000000     −2.44979100000000
Ni  0.02873500000000      0.04071200000000      4.17666200000000
O   0.14010700000000      2.12495900000000      4.20327400000000
O  −0.08263700000000     −2.04353500000000      4.15005100000000
O   0.02463200000000      0.01428200000000      6.26388100000000
O   2.11314800000000     −0.07070300000000      4.17934900000000
O  −2.05567800000000      0.15212800000000      4.17397500000000
H   1.08187800000000      2.34074500000000      4.34112800000000
H   0.05055700000000      2.03724900000000      3.21956000000000
H  −0.84478000000000     −2.42371700000000      3.67878900000000
H  −0.30075900000000     −2.03755900000000      5.11440900000000
H   2.00987000000000     −0.26205800000000      5.14841500000000
H   2.30538800000000     −0.93026400000000      3.76085000000000
H  −2.02164000000000      0.14815200000000      5.16421000000000
H  −2.57718300000000     −0.62814100000000      3.91746800000000
Ni  0.02052800000000     −0.01214800000000      8.35110000000000
O   0.13190100000000      2.07209900000000      8.37771100000000
O  −0.09084400000000     −2.09639500000000      8.32448800000000
O  −2.06388400000000      0.09926800000000      8.34841300000000
O   2.10494100000000     −0.12356300000000      8.35378700000000
O   0.01642500000000     −0.03857800000000     10.43831800000000
H  −0.54404800000000      2.59824300000000      8.83883900000000
H  −0.02409600000000      2.16166300000000      7.40756400000000
H   0.79793500000000     −2.38740200000000      8.60424700000000
H  −0.02755200000000     −1.96685100000000      7.34068400000000
H  −2.41721400000000      0.95350100000000      8.04262800000000
H  −1.97217200000000      0.16346900000000      9.33395400000000
H   2.38466700000000      0.71248000000000      8.77157900000000
H   2.04281500000000      0.06940700000000      7.38311000000000
Ni  0.01232200000000     −0.06500800000000     12.52553700000000
O   0.12369500000000      2.01923900000000     12.55214800000000
O  −0.09905000000000     −2.14925500000000     12.49892600000000
O  −2.07209100000000      0.04640700000000     12.52285000000000
O   2.09673500000000     −0.17642400000000     12.52822400000000
O   0.00821900000000     −0.09143800000000     14.61275600000000
H   0.18414300000000      0.74582900000000     15.07853100000000
H   0.63714700000000     −0.73845000000000     14.98280400000000
H   0.12170700000000      1.87545100000000     11.55963200000000
H  −0.74610800000000      2.41427500000000     12.75483800000000
H  −0.89278600000000     −2.64417300000000     12.77005100000000
H  −0.11720000000000     −2.10178600000000     11.50772600000000
H  −2.63365300000000     −0.60027200000000     12.98762300000000
H  −2.05402400000000     −0.18737500000000     11.56160400000000
H   2.51568100000000      0.68694800000000     12.70603100000000
H   2.06785300000000     −0.25691100000000     11.54147900000000
```

v. n=5 Ni:

```
Ni  0.12893400000000      0.03779500000000      0.00112800000000
O   2.21517600000000     −0.02364300000000      0.03304300000000
O  −1.95730700000000      0.09923200000000     −0.03078800000000
O   0.19021900000000      2.12425900000000      0.01153000000000
O   0.06764900000000     −2.04867000000000     −0.00927500000000
O   0.16114200000000      0.04725500000000     −2.08599300000000
O   0.09672700000000      0.02833500000000      2.08824800000000
H   2.08131100000000     −0.01020000000000      1.02236000000000
H   2.52119700000000     −0.93036300000000     −0.16287700000000
H  −0.68844700000000      2.51529500000000     −0.15503000000000
H   0.24299300000000      2.02334900000000      1.00223900000000
H  −1.82524200000000      0.08916400000000      0.96422000000000
H  −2.48394900000000     −0.69128300000000     −0.24736600000000
H  −0.09252700000000     −2.02488800000000      0.96929100000000
H  −0.61807700000000     −2.59883300000000     −0.42703400000000
H  −0.56607700000000      0.51881600000000     −2.53059200000000
H   0.97973400000000      0.46068400000000     −2.41845300000000
Ni  0.06451900000000      0.01887600000000      4.17536800000000
O   0.12580400000000      2.10534000000000      4.18577000000000
O   0.00323400000000     −2.06758800000000      4.16496600000000
O   0.03231100000000      0.00941700000000      6.26248800000000
O   2.15076000000000     −0.04256100000000      4.20728300000000
O  −2.02172300000000      0.08031300000000      4.14345200000000
H  −0.70220500000000      2.33566200000000      3.72381100000000
H  −0.10615000000000      2.03716200000000      5.14677800000000
H   0.19136400000000     −2.05736600000000      5.13593900000000
H   0.84852100000000     −2.28546400000000      3.72904300000000
H   2.08295600000000      0.18037800000000      5.16956300000000
H   2.39784300000000      0.78585400000000      3.75432800000000
H  −2.00806700000000     −0.10485800000000      5.11589800000000
H  −2.24128500000000     −0.76728300000000      3.71423600000000
Ni  0.00010400000000     −0.00004300000000      8.34960800000000
O   0.06138900000000      2.08642100000000      8.36001000000000
O  −0.06118100000000     −2.08650700000000      8.33920600000000
O  −2.08613800000000      0.06139500000000      8.31769200000000
O   2.08634500000000     −0.06148000000000      8.38152300000000
O  −0.03210400000000     −0.00950200000000     10.43672800000000
H   0.87059100000000      2.26962600000000      8.87358200000000
H   0.34380700000000      2.03106200000000      7.41367500000000
H  −0.34335900000000     −2.02929800000000      9.28560600000000
H  −0.87078900000000     −2.26910000000000      7.82604600000000
H  −2.31156900000000      0.88347100000000      7.84423000000000
H  −2.01500500000000      0.31207900000000      9.27358900000000
H   2.31002400000000     −0.88498100000000      8.85333600000000
H   2.01318500000000     −0.31083500000000      7.42530300000000
Ni −0.06431100000000     −0.01896200000000     12.52384800000000
O  −0.00302700000000      2.06750300000000     12.53425000000000
O  −0.12559600000000     −2.10542600000000     12.51344600000000
O  −2.15055300000000      0.04247600000000     12.49193200000000
O   2.02193000000000     −0.08039900000000     12.55576400000000
O  −0.09651900000000     −0.02842100000000     14.61096800000000
H  −0.18926400000000      2.05556500000000     11.56284400000000
H  −0.84912300000000      2.28637800000000     12.96806500000000
H   0.70138800000000     −2.34020800000000     12.97489600000000
H   0.10583300000000     −2.03894500000000     11.55227100000000
H  −2.39571000000000     −0.78663200000000     12.94469800000000
H  −2.08109600000000     −0.18030100000000     11.52962500000000
H   2.24348600000000      0.76660900000000     12.98504200000000
H   2.00978800000000      0.10449100000000     11.58334900000000
Ni −0.12872600000000     −0.03788000000000     16.69808800000000
O  −0.06744200000000      2.04858400000000     16.70849000000000
O  −0.19001100000000     −2.12434400000000     16.68768600000000
O  −2.21496800000000      0.02355700000000     16.66617300000000
O   1.95751500000000     −0.09931700000000     16.73000400000000
O  −0.16093400000000     −0.04733900000000     18.78520800000000
H  −0.98005400000000     −0.45975500000000     19.11763600000000
H   0.56598000000000     −0.51904400000000     19.23016800000000
H   0.61526100000000      2.60188500000000     17.12707200000000
H   0.09279200000000      2.02666700000000     15.72999600000000
H   0.68864000000000     −2.51535500000000     16.85440400000000
H  −0.24186700000000     −2.02249800000000     15.69692900000000
H  −2.52146100000000      0.93005900000000     16.86239400000000
H  −2.08197600000000      0.01010500000000     15.67678400000000
H   1.82176200000000     −0.08850900000000     15.73511600000000
H   2.48162500000000      0.69296400000000     16.94614599999999
```

vi. n=6 Ni:

```
Ni −0.08614600000000      0.07092100000000      0.00057800000000
O   2.00118500000000      0.07094800000000     −0.01512100000000
O  −2.17347700000000      0.07089500000000      0.01627700000000
O  −0.08605400000000      2.15825200000000      0.01628700000000
O  −0.08623700000000     −2.01641000000000     −0.01513000000000
O  −0.10184500000000      0.08663000000000     −2.08669400000000
O  −0.07044700000000      0.05521200000000      2.08785000000000
H   1.84846900000000      0.06862600000000      0.97873400000000
H   2.50589500000000     −0.73767500000000     −0.21614800000000
H   0.80877900000000      2.51907600000000     −0.13096800000000
H  −0.15376100000000      2.04347800000000      1.00626800000000
H  −2.03871200000000      0.08143100000000      1.00674400000000
H  −2.50052700000000     −0.82883200000000     −0.17745600000000
H   0.04963000000000     −1.97439900000000      0.96892500000000
H   0.60813800000000     −2.57890400000000     −0.40080600000000
H  −0.90675200000000      0.52878500000000     −2.41543000000000
H   0.64053000000000      0.55086500000000     −2.51348000000000
Ni −0.05474700000000      0.03950400000000      4.17512200000000
O  −0.05465600000000      2.12683500000000      4.19083000000000
O  −0.05483900000000     −2.04782700000000      4.15941300000000
O  −0.03904800000000      0.02379500000000      6.26239400000000
O   2.03258400000000      0.03953000000000      4.15942200000000
O  −2.14207800000000      0.03947700000000      4.19082100000000
H   0.11106700000000      2.04166000000000      5.16730700000000
H   0.80846600000000      2.33903500000000      3.78970700000000
H  −0.92479600000000     −2.24413100000000      3.76418100000000
H  −0.21059900000000     −1.97384500000000      5.13901200000000
H   1.98068800000000     −0.12466100000000      5.13667400000000
H   2.22492500000000     −0.82697100000000      3.75580600000000
H  −2.03965200000000      0.22586200000000      5.16124200000000
H  −2.35358500000000      0.89507800000000      3.77190100000000
Ni −0.02334900000000      0.00808700000000      8.34966500000000
O  −0.02325700000000      2.09541800000000      8.36537400000000
O  −0.02344100000000     −2.07924400000000      8.33395700000000
O  −2.11068000000000      0.00806000000000      8.36536500000000
O   2.06398200000000      0.00811300000000      8.33396600000000
O  −0.00765000000000     −0.00762200000000     10.43693700000000
H  −0.28693500000000      1.99832300000000      9.31637900000000
H  −0.83453000000000      2.34108600000000      7.88342400000000
H   0.23682800000000     −2.07214100000000      9.28713300000000
H   0.78963300000000     −2.26415600000000      7.82779500000000
H  −2.00560800000000     −0.23180400000000      7.40688600000000
H  −2.35734000000000     −0.81860300000000      8.81924300000000
H   1.99438200000000      0.23268700000000      9.29713300000000
H   2.24727200000000      0.84880000000000      7.87370500000000
Ni  0.00804900000000     −0.02333100000000     12.52420900000000
O   0.00814100000000      2.06400000000000     12.53991700000000
O   0.00795800000000     −2.11066100000000     12.50850100000000
O  −2.07928200000000     −0.02335700000000     12.53990900000000
O   2.09538000000000     −0.02330400000000     12.50851000000000
O   0.02374800000000     −0.03903900000000     14.61148100000000
H   0.84847800000000      2.24771800000000     13.00063600000000
H   0.23304800000000      1.99529100000000     11.57682200000000
H  −0.81850500000000     −2.35788200000000     12.05457100000000
H  −0.23210600000000     −2.00571400000000     13.46694100000000
H  −2.26364100000000      0.78972300000000     13.04626900000000
H  −2.07188800000000      0.23722100000000     11.58681400000000
H   2.34071200000000     −0.83568000000000     12.98877800000000
H   1.99772600000000     −0.28533100000000     11.55704900000000
Ni  0.03944800000000     −0.05474800000000     16.69875300000000
O   0.03953900000000      2.03258300000000     16.71446100000000
O   0.03935600000000     −2.14207900000000     16.68304500000000
O  −2.04788300000000     −0.05477400000000     16.71445200000000
O   2.12677900000000     −0.05472100000000     16.68305300000000
O   0.05514700000000     −0.07045600000000     18.78602500000000
H  −0.82722500000000      2.22389500000000     17.11805099999999
H  −0.12446700000000      1.98069100000000     15.73717000000000
H   0.89472400000000     −2.35342600000000     17.10254600000000
H   0.22640600000000     −2.04031200000000     15.71272600000000
H  −1.97338700000000     −0.21040900000000     15.73482600000000
H  −2.24227200000000     −0.92520300000000     17.10965000000000
H   2.33823800000000      0.80781100000000     17.08588700000000
H   2.04354800000000      0.11217700000000     15.70675600000000
Ni  0.07084600000000     −0.08616500000000     20.87329700000000
O   0.07093800000000      2.00116600000000     20.88900500000000
O   0.07075400000000     −2.17349600000000     20.85758800000000
O  −2.01648500000000     −0.08619200000000     20.88899599999999
O   2.15817700000000     −0.08613800000000     20.85759699999999
O   0.08654600000000     −0.10187400000000     22.96056800000000
H   0.55080900000000      0.64038200000000     23.38751300000000
H   0.52851100000000     −0.90686300000000     23.28933400000000
H   0.06861200000000      1.84824100000000     19.89516800000000
H  −0.73747900000000      2.50620600000000     21.08997000000000
H  −0.82895500000000     −2.50064000000000     21.05119600000000
H   0.08123600000000     −2.03792300000000     19.86717800000000
H  −2.57827200000000      0.60866900000000     21.27480300000000
H  −1.97441300000000      0.04982900000000     19.90498100000000
H   2.51878400000000      0.80880500000000     21.00467300000000
H   2.04291700000000     −0.15393100000000     19.86765300000000
```

vii. n=7 Ni:

```
Ni −0.06894700000000      0.12001500000000     −0.00106500000000
O   2.01841500000000      0.12609000000000     −0.00995100000000
O  −2.15630900000000      0.11394100000000      0.00782100000000
O  −0.07493500000000      2.20730000000000      0.01908600000000
O  −0.06295800000000     −1.96726900000000     −0.02121700000000
O  −0.07789100000000      0.14014100000000     −2.08833900000000
O  −0.06000200000000      0.09989000000000      2.08620900000000
H   1.83730500000000      0.13348500000000      0.97972900000000
H   2.35189400000000     −0.77186700000000     −0.19784300000000
H   0.81662900000000      2.58000800000000     −0.11821900000000
H  −0.14220400000000      2.06002800000000      1.00352400000000
H  −2.07704600000000      0.16478300000000      0.99924300000000
H  −2.67460500000000     −0.68782500000000     −0.18246300000000
H  −0.21827600000000     −1.91269100000000      0.95798300000000
H  −0.78082300000000     −2.49621700000000     −0.41176300000000
H  −0.71197500000000      0.80867200000000     −2.40812500000000
H   0.77415200000000      0.36650900000000     −2.50257800000000
Ni −0.05105800000000      0.07976400000000      4.17348200000000
O  −0.05704600000000      2.16704800000000      4.19363400000000
O  −0.04507000000000     −2.00752000000000      4.15333100000000
O  −0.04211400000000      0.05963900000000      6.26075600000000
O   2.03630400000000      0.08583900000000      4.16459600000000
O  −2.13842000000000      0.07369000000000      4.18236900000000
H   0.85098700000000      2.42599700000000      4.43619200000000
H  −0.04339200000000      2.06623600000000      3.20661000000000
H   0.01165600000000     −1.90743600000000      5.14713200000000
H   0.76777100000000     −2.45710900000000      3.86448900000000
H   1.98182800000000     −0.06420500000000      5.14626200000000
H   2.49226100000000     −0.67948300000000      3.77466500000000
H  −2.00995400000000      0.17072600000000      5.16537600000000
H  −2.37392400000000     −0.86355400000000      4.05081400000000
Ni −0.03316900000000      0.03951300000000      8.34803000000000
O  −0.03915800000000      2.12679800000000      8.36818100000000
O  −0.02718100000000     −2.04777100000000      8.32787900000000
O  −2.12053100000000      0.03343900000000      8.35691600000000
O   2.05419300000000      0.04558800000000      8.33914400000000
O  −0.02422500000000      0.01938800000000     10.43530400000000
H  −0.85108500000000      2.29706000000000      8.88118700000000
H  −0.30260300000000      2.13059900000000      7.41536100000000
H   0.14541600000000     −1.97237000000000      9.30501800000000
H   0.83997500000000     −2.22099500000000      7.91701500000000
H  −2.04273900000000     −0.25386000000000      7.41249200000000
H  −2.34236500000000     −0.76416700000000      8.87148700000000
H   1.93174600000000      0.17873100000000      9.32128000000000
H   2.27269100000000      0.92825300000000      7.98808600000000
Ni −0.01528000000000     −0.00073700000000     12.52257800000000
O  −0.02126900000000      2.08654700000000     12.54272900000000
O  −0.00929200000000     −2.08802200000000     12.50242700000000
O  −2.10264300000000     −0.00681200000000     12.53146400000000
O   2.07208200000000      0.00533700000000     12.51369100000000
O  −0.00633600000000     −0.02086300000000     14.60985200000000
H   0.82502700000000      2.31468400000000     12.96863800000000
H   0.17786000000000      2.00826500000000     11.57177400000000
H  −0.86152400000000     −2.31757500000000     12.08933200000000
H  −0.19461800000000     −2.01402000000000     13.47660800000000
H  −2.27654200000000      0.81754000000000     13.02312300000000
H  −2.04245200000000      0.24242300000000     11.57307200000000
H   2.00441200000000     −0.24187400000000     13.47270100000000
H   2.24330600000000     −0.82140400000000     12.02505700000000
Ni  0.00260900000000     −0.04098800000000     16.69712500000000
O  −0.00338000000000      2.04629600000000     16.71727700000000
O   0.00859700000000     −2.12827200000000     16.67697400000000
O  −2.08475400000000     −0.04706300000000     16.70601200000000
O   2.08997100000000     −0.03491400000000     16.68823900000000
O   0.01155300000000     −0.06111400000000     18.78439900000000
H  −0.87245100000000      2.21783000000000     17.12514700000000
H  −0.17490000000000      1.96565500000000     15.73985000000000
H   0.17022600000000     −2.10247800000000     17.65397500000000
H   0.87091900000000     −2.30839900000000     16.25908400000000
H  −1.96799300000000     −0.19081200000000     15.72542800000000
H  −2.32401300000000     −0.92030000000000     17.06759700000000
H   2.01291200000000      0.27659100000000     17.62465400000000
H   2.30266100000000      0.74881600000000     16.14869800000000
Ni  0.02049800000000     −0.08123900000000     20.87167300000000
O   0.01450900000000      2.00604500000000     20.89182400000000
O   0.02648600000000     −2.16852300000000     20.85152200000000
O  −2.06686500000000     −0.08731300000000     20.88055900000000
O   2.10786000000000     −0.07516500000000     20.86278699999999
O   0.02944200000000     −0.10136400000000     22.95894700000000
H  −0.05358700000000      1.91363100000000     19.89895599999999
H  −0.80321900000000      2.43511100000000     21.19745099999999
H  −0.00080600000000     −2.06422100000000     21.83833300000000
H   0.94627900000000     −2.43027200000000     20.65925199999999
H  −2.52866700000000      0.74238300000000     21.09160600000000
H  −1.98193500000000     −0.11028100000000     19.88924900000000
H   2.32927900000000      0.81778600000000     21.18572400000000
H   1.95950800000000      0.02207500000000     19.87908400000000
Ni  0.03838600000000     −0.12149000000000     25.04622099999999
O   0.03239800000000      1.96579400000000     25.06637200000000
O   0.04437500000000     −2.20877400000000     25.02607000000000
O  −2.04897600000000     −0.12756400000000     25.05510700000000
O   2.12574900000000     −0.11541500000000     25.03733499999999
O   0.04733100000000     −0.14161500000000     27.13349400000000
H   0.74548700000000      2.50519000000000     25.45136800000000
H   0.17856100000000      1.91361300000000     24.08582199999999
H  −0.84524500000000     −2.58317600000000     25.17301300000000
H   0.09409800000000     −2.05420700000000     24.04120000000000
H  −2.39175800000000      0.76760500000000     25.23879799999999
H  −1.89889800000000     −0.14792800000000     24.06347400000000
H   2.01539100000000     −0.15083200000000     24.04612500000000
H   2.63001100000000      0.69552700000000     25.22712200000000
H  −0.80752800000000     −0.35811000000000     27.54730600000000
H   0.67392800000000     −0.81643400000000     27.45460100000000
```

viii. n=8 Ni:

```
Ni −0.01795400000000    0.00353200000000    12.52449000000000
O  −0.01310100000000    2.09088900000000    12.53519000000000
O  −0.02280800000000   −2.08382500000000    12.51379000000000
O  −2.10531100000000    0.00833000000000    12.53525300000000
O   2.06940300000000   −0.00126600000000    12.51372600000000
O  −0.00721500000000   −0.00719300000000    14.61182400000000
H   0.75643500000000    2.29613700000000    13.09794100000000
H   0.32458400000000    2.02875300000000    11.60721500000000
H  −0.87828800000000   −2.29455800000000    12.09658700000000
H  −0.21624500000000   −1.97809500000000    13.48459200000000
H  −2.23075900000000    0.74245200000000    13.16737800000000
H  −2.08489900000000    0.40671100000000    11.63336000000000
H   1.98322900000000   −0.21241200000000    13.48145600000000
H   2.24910900000000   −0.84855900000000    12.06506700000000
Ni  0.00352400000000   −0.01791800000000    16.69915900000000
O   0.00837700000000    2.06943900000000    16.70985900000000
O  −0.00133000000000   −2.10527400000000    16.68845900000000
O  −2.08383300000000   −0.01311900000000    16.70992300000000
O   2.09088000000000   −0.02271600000000    16.68839600000000
O   0.01426200000000   −0.02864200000000    18.78649400000000
H  −0.83807400000000    2.25355500000000    17.15831700000000
H  −0.20282500000000    1.98489700000000    15.74202800000000
H   0.40015000000000   −2.08796000000000    17.58895700000000
H   0.73005800000000   −2.23228800000000    16.05341500000000
H  −1.97921000000000   −0.20641400000000    15.73899100000000
H  −2.29609600000000   −0.86835100000000    17.12689800000000
H   2.02996300000000    0.31603400000000    17.61603599999999
H   2.29914500000000    0.74546100000000    16.12491700000000
Ni  0.02500100000000   −0.03936700000000    20.87382900000000
O   0.02985500000000    2.04799000000000    20.88452899999999
O   0.02014800000000   −2.12672400000000    20.86312900000000
O  −2.06235500000000   −0.03456900000000    20.88459200000000
O   2.11235800000000   −0.04416500000000    20.86306499999999
O   0.03574000000000   −0.05009200000000    22.96116400000000
H   0.86945700000000    2.20340900000000    21.35639099999999
H   0.25325300000000    2.01032700000000    19.92058900000000
H  −0.27391700000000   −2.05325400000000    19.92054600000000
H  −0.78383200000000   −2.27835200000000    21.39567900000000
H  −2.21217100000000    0.78763300000000    21.38815000000000
H  −2.04146100000000    0.21880500000000    19.92903200000000
H   2.26985500000000   −0.86581500000000    21.36580500000000
H   2.02644800000000   −0.30911000000000    19.91160200000000
Ni  0.04647900000000   −0.06081700000000    25.04849900000000
O   0.05133200000000    2.02654000000000    25.05919800000000
O   0.04162500000000   −2.14817400000000    25.03779900000000
O  −2.04087800000000   −0.05601800000000    25.05926200000000
O   2.13383600000000   −0.06561500000000    25.03773500000000
O   0.05721700000000   −0.07154100000000    27.13583300000000
H  −0.83170700000000    2.21572000000000    25.42672300000000
H  −0.08452900000000    1.91677800000000    24.07623399999999
H   0.91166900000000   −2.35490400000000    25.42744899999999
H   0.19589300000000   −2.04328500000000    24.05918899999999
H  −1.93478200000000   −0.19860100000000    24.07762599999999
H  −2.22657500000000   −0.93517900000000    25.43885600000000
H   2.33643600000000    0.80886200000000    25.41863299999999
H   2.02506200000000    0.08189000000000    24.05787300000000
Ni −0.08238700000000    0.06788100000000     0.00048100000000
O   2.00497000000000    0.06308200000000    −0.01028300000000
O  −2.16974400000000    0.07267900000000     0.01124400000000
O  −0.07753400000000    2.15523800000000     0.01118100000000
O  −0.08724000000000   −2.01947600000000    −0.01021900000000
O  −0.09312600000000    0.07860600000000    −2.08685400000000
O  −0.07164800000000    0.05715600000000     2.08781500000000
H   1.83476700000000    0.06593600000000     0.98239100000000
H   2.51256700000000   −0.74748100000000    −0.19538600000000
H   0.82240200000000    2.50964100000000    −0.11886100000000
H  −0.15367600000000    2.02437800000000     1.00013600000000
H  −2.02224400000000    0.09177900000000     1.00125800000000
H  −2.49304600000000   −0.83107000000000    −0.16855600000000
H   0.02927400000000   −1.94241200000000     0.97698200000000
H   0.63170200000000   −2.57510800000000    −0.35889900000000
H  −0.90018000000000    0.52773800000000    −2.40046400000000
H   0.64624600000000    0.56358400000000    −2.49527200000000
Ni −0.06090900000000    0.04643100000000     4.17515000000000
O  −0.05605600000000    2.13378800000000     4.18585000000000
O  −0.06576300000000   −2.04092600000000     4.16445000000000
O  −0.05017100000000    0.03570600000000     6.26248500000000
O   2.02644700000000    0.04163300000000     4.16438700000000
O  −2.14826600000000    0.05122900000000     4.18591400000000
H   0.09311800000000    2.02597500000000     5.16538300000000
H   0.81859600000000    2.33093300000000     3.80243000000000
H  −0.94551500000000   −2.22270800000000     3.78437000000000
H  −0.20807600000000   −1.93539000000000     5.14610200000000
H   1.91552600000000   −0.09374500000000     5.14734000000000
H   2.21107600000000   −0.84236500000000     3.79684200000000
H  −2.04233000000000    0.20542700000000     5.16441900000000
H  −2.35134200000000    0.92202200000000     3.79604400000000
Ni −0.03943200000000    0.02498200000000     8.34982000000000
O  −0.03457800000000    2.11233900000000     8.36052000000000
O  −0.04428500000000   −2.06237500000000     8.33912000000000
O  −2.12678800000000    0.02978000000000     8.36058400000000
O   2.04792500000000    0.02018300000000     8.33905600000000
O  −0.02869300000000    0.01425700000000    10.43715500000000
H  −0.29947800000000    2.02660600000000     9.31205100000000
H  −0.85574500000000    2.27259700000000     7.85786300000000
H   0.20881500000000   −2.04243200000000     9.29478600000000
H   0.77773300000000   −2.21426500000000     7.83585700000000
H  −2.28277600000000   −0.77326600000000     7.82789900000000
H  −2.05619300000000   −0.26435900000000     9.30328000000000
H   2.01335800000000    0.24441800000000     9.30277900000000
H   2.20627400000000    0.85869000000000     7.86619800000000
Ni  0.06795600000000   −0.08226600000000    29.22316800000000
O   0.07280900000000    2.00509100000000    29.23386800000000
O   0.06310200000000   −2.16962300000000    29.21246799999999
O  −2.01940100000000   −0.07746800000000    29.23393099999999
O   2.15531300000000   −0.08706400000000    29.21240500000000
O   0.07869400000000   −0.09299100000000    31.31050299999999
H   0.07467600000000    1.83484600000000    28.24122400000000
H  −0.73539200000000    2.51627300000000    29.41946000000000
H  −0.84260900000000   −2.48784200000000    29.39153499999999
H   0.08404700000000   −2.02266900000000    28.22244900000000
H  −2.57220900000000    0.64463300000000    29.58055200000000
H  −1.94004500000000    0.03701100000000    28.24648000000000
H   2.51430500000000    0.81082300000000    29.34400800000000
H   2.02376900000000   −0.16092000000000    28.22328699999999
H   0.56416600000000    0.64568600000000    31.71961700000000
H   0.52661800000000   −0.90081600000000    31.62390600000000
```

ix. n=9 Ni:

```
Ni -0.08688900000000      0.10264100000000     -0.00183700000000
O   2.00046900000000      0.10729600000000     -0.01237200000000
O  -2.17424700000000      0.09798500000000      0.00869800000000
O  -0.09148900000000      2.18999700000000      0.00922000000000
O  -0.08228900000000     -1.98471500000000     -0.01289400000000
O  -0.09744900000000      0.11367400000000     -2.08917100000000
O  -0.07633000000000      0.09160700000000      2.08549700000000
H   1.80653100000000      0.11810800000000      0.97568700000000
H   2.33717200000000     -0.79097600000000     -0.19130600000000
H   0.80765800000000      2.54943500000000     -0.11232800000000
H  -0.16984900000000      2.03226100000000      0.99231900000000
H  -2.08002400000000      0.15263000000000      0.99924200000000
H  -2.70940500000000     -0.69571900000000     -0.16843000000000
H  -0.22277600000000     -1.89975100000000      0.96819800000000
H  -0.82501500000000     -2.49868100000000     -0.37572100000000
H  -0.72512400000000      0.79241700000000     -2.39961600000000
H   0.75895400000000      0.34618300000000     -2.49061700000000
Ni -0.06577000000000      0.08057400000000      4.17283200000000
O  -0.07037000000000      2.16793000000000      4.18388800000000
O  -0.06117000000000     -2.00678200000000      4.16177500000000
O  -0.05521100000000      0.06954000000000      6.26016600000000
O   2.02158800000000      0.08522900000000      4.16229700000000
O  -2.15312800000000      0.07591800000000      4.18336700000000
H   0.82893700000000      2.41941400000000      4.46370200000000
H  -0.01906200000000      2.06167300000000      3.19868200000000
H  -0.00440800000000     -1.88800700000000      5.15553100000000
H   0.75366100000000     -2.45782200000000      3.88092000000000
H   1.95208800000000     -0.05993800000000      5.14530900000000
H   2.46433000000000     -0.69200100000000      3.78086600000000
H  -2.01497600000000      0.17577800000000      5.16592100000000
H  -2.38384300000000     -0.86316100000000      4.05677400000000
Ni -0.04465100000000      0.05850700000000      8.34750000000000
O  -0.04925100000000      2.14586300000000      8.35855700000000
O  -0.04005100000000     -2.02884900000000      8.33644300000000
O  -2.13200900000000      0.05385100000000      8.35803500000000
O   2.04270700000000      0.06316200000000      8.33696500000000
O  -0.03409200000000      0.04747300000000     10.43483400000000
H  -0.84297800000000      2.29546000000000      8.90585900000000
H  -0.34876300000000      2.14156800000000      7.41688200000000
Ni -0.02353200000000      0.03644000000000     12.52216800000000
O  -0.02813200000000      2.12379600000000     12.53322500000000
O  -0.01893200000000     -2.05091600000000     12.51111100000000
O  -2.11089000000000      0.03178400000000     12.53270300000000
O   2.06382600000000      0.04109600000000     12.51163300000000
O  -0.01297300000000      0.02540600000000     14.60950200000000
H   0.81549900000000      2.34361000000000     12.96874300000000
H   0.18095200000000      2.02866600000000     11.56484000000000
H  -0.87483800000000     -2.26424200000000     12.09681600000000
H  -0.20656400000000     -1.95305000000000     13.48411600000000
H  -2.26223300000000      0.84896500000000     13.04414700000000
H  -2.04240600000000      0.29986500000000     11.57971500000000
H   1.99573900000000     -0.19441400000000     13.47411400000000
H   2.22181700000000     -0.79335800000000     12.03168200000000
Ni -0.00241300000000      0.01437300000000     16.69683600000000
O  -0.00701300000000      2.10172900000000     16.70789300000000
O   0.00218700000000     -2.07298300000000     16.68577900000000
O  -2.08977100000000      0.00971700000000     16.70737100000000
O   2.08494500000000      0.01902900000000     16.68630100000000
O   0.00814600000000      0.00334000000000     18.78417000000000
H  -0.83193900000000      2.25468600000000     17.20604300000000
H  -0.25901700000000      2.04548500000000     15.75005300000000
H   0.25369500000000     -2.01659000000000     17.64378400000000
H   0.82763300000000     -2.22429100000000     16.18796600000000
H  -1.99167500000000     -0.18446100000000     15.73570400000000
H  -2.30628500000000     -0.84161500000000     17.12929199999999
H   1.98686400000000      0.21085800000000     17.65850900000000
H   2.30095200000000      0.87164600000000     16.26675100000000
Ni  0.01870600000000     -0.00769400000000     20.87150500000000
O   0.01410600000000      2.07966200000000     20.88256100000000
O   0.02330600000000     -2.09505000000000     20.86044799999999
O  -2.06865200000000     -0.01235000000000     20.88204000000000
O   2.10606400000000     -0.00303800000000     20.86097000000000
O   0.02926500000000     -0.01872700000000     22.95883900000000
H   0.86802300000000      2.29375200000000     21.30050600000000
H   0.20467900000000      1.98408200000000     19.91013300000000
H  -0.82312800000000     -2.31291200000000     20.42939600000000
H  -0.18170600000000     -1.99697600000000     21.82977300000000
H  -2.22758600000000      0.81877200000000     21.36751200000000
H  -2.00342800000000      0.22845400000000     19.92098099999999
H   2.03584800000000     -0.26201700000000     21.81673700000000
H   2.25890800000000     -0.82520800000000     20.35816000000000
Ni  0.03982500000000     -0.02976100000000     25.04617300000000
O   0.03522500000000      2.05759500000000     25.05723000000000
O   0.04442500000000     -2.11711700000000     25.03511600000000
O  -2.04753300000000     -0.03441600000000     25.05670800000000
O   2.12718300000000     -0.02510500000000     25.03563800000000
O   0.05038400000000     -0.04079400000000     27.13350699999999
H  -0.82387500000000      2.22275400000000     25.48872500000000
H  -0.15552300000000      1.96755900000000     24.08379699999999
H   0.33501600000000     -2.11044700000000     25.97994600000000
H   0.84328100000000     -2.26628700000000     24.49523900000000
H  -1.91914200000000     -0.17408900000000     24.07571000000000
H  -2.26125300000000     -0.91570000000000     25.41389100000000
H   2.02449400000000      0.27598900000000     25.97501900000000
H   2.31890700000000      0.77014900000000     24.50506300000000
Ni  0.06094400000000     -0.05182800000000     29.22084099999999
O   0.05634400000000      2.03552800000000     29.23189800000000
O   0.06554400000000     -2.13918300000000     29.20978400000000
O  -2.02641400000000     -0.05648300000000     29.23137599999999
O   2.14830200000000     -0.04717200000000     29.21030599999999
O   0.07150300000000     -0.06286100000000     31.308174999999994
H   0.12670800000000     -1.92758700000000      9.31457300000000
H   0.83289800000000     -2.19175000000000      7.93361800000000
H  -2.06152700000000     -0.28759900000000      7.43237000000000
H  -2.32693600000000     -0.71296300000000      8.92806600000000
H   1.91101400000000      0.19947400000000      9.31854800000000
H   2.24595000000000      0.94818600000000      7.98253500000000
H   0.85323900000000      2.46125100000000     29.59253799999999
H   0.18257500000000      1.94584100000000     28.24505900000000
H  -0.84984500000000     -2.37507700000000     28.97133699999999
H   0.05704400000000     -2.01659600000000     30.19569600000000
H  -2.22744100000000      0.81012500000000     29.63071800000000
H   2.60191300000000      0.80682600000000     29.32087900000000
H   1.99808700000000     -0.15107400000000     28.22734600000000
Ni  0.08206300000000     -0.07389400000000     33.39550900000000
O   0.07746300000000      2.01346200000000     33.40656600000000
H  -1.94614800000000      0.10580100000000     28.25277000000000
O   0.08666300000000     -2.16124900000000     33.38445300000000
O  -2.00529500000000     -0.07854900000000     33.40604500000000
O   2.16942100000000     -0.06923800000000     33.38497400000000
O   0.09262300000000     -0.08492600000000     35.48284400000000
H  -0.81139500000000      2.37779000000000     33.57780700000000
```

```
H   0.10149100000000      1.87842800000000     32.41814900000000
H  −0.63007800000000     −2.73779000000000     33.70323600000000
H  −0.01919700000000     −2.05188700000000     32.40151000000000
H  −2.52148300000000     −0.88084100000000     33.60362300000000
H  −1.82964700000000     −0.09965600000000     32.41612600000000
H   2.49755100000000     −0.97120300000000     33.56434700000000
H   2.03618600000000     −0.04975300000000     32.39548200000000
H   0.89676500000000      0.36773600000000     35.79837000000000
H  −0.64981800000000      0.39328800000000     35.89328200000000
```

x. n=10 Ni:

```
Ni −0.08067600000000     −0.03164800000000     −0.05728200000000
O   2.00656300000000     −0.05422400000000     −0.06839200000000
O  −2.16791400000000     −0.00907300000000     −0.04617200000000
O  −0.05811700000000      2.05561700000000     −0.06041500000000
O  −0.10323400000000     −2.11891400000000     −0.05415100000000
O  −0.09181900000000     −0.03466000000000     −2.14464000000000
O  −0.06953300000000     −0.02863700000000      2.03007600000000
H   2.52074600000000      0.75502800000000     −0.24047700000000
H   1.82096900000000     −0.05898700000000      0.92310400000000
H   0.67937700000000      2.60479800000000     −0.37936900000000
H   0.04218400000000      1.94960000000000      0.92777800000000
H  −2.47517300000000      0.90239200000000     −0.21395900000000
H  −2.00821200000000     −0.03791700000000      0.94253700000000
H  −0.17808000000000     −1.97441000000000      0.93414400000000
H   0.79437600000000     −2.48172500000000     −0.17609200000000
H  −0.90437900000000     −0.48294100000000     −2.44500400000000
H   0.64091600000000     −0.54056900000000     −2.53935600000000
Ni −0.05839000000000     −0.02562500000000      4.11743400000000
O  −0.03583100000000      2.06164100000000      4.11430200000000
O  −0.08094800000000     −2.11289100000000      4.12056600000000
O  −0.04724600000000     −0.02261300000000      6.20479200000000
O   2.02884900000000     −0.04820000000000      4.10632400000000
O  −2.14562800000000     −0.00305000000000      4.12854400000000
H  −0.17506600000000      1.94872900000000      5.09648800000000
H  −0.91672700000000      2.24695900000000      3.73868900000000
H   0.79552900000000     −2.31282700000000      3.74305800000000
H   0.06290100000000     −2.00303400000000      5.10191900000000
H   1.90448500000000      0.07742000000000      5.09099100000000
H   2.21724900000000      0.83969500000000      3.75053700000000
H  −2.03251300000000     −0.14997100000000      5.10884200000000
H  −2.35274000000000     −0.87648000000000      3.74712400000000
Ni −0.03610300000000     −0.01960200000000      8.29215000000000
O  −0.01354500000000      2.06766400000000      8.28901800000000
O  −0.05866200000000     −2.10686800000000      8.29528200000000
O  −2.12334200000000      0.00297300000000      8.30326000000000
O   2.05113500000000     −0.04217700000000      8.28104000000000
O  −0.02496000000000     −0.01659000000000     10.37950800000000
H   0.19221300000000      1.98019900000000      9.25792300000000
H   0.83939700000000      2.20950900000000      7.83731000000000
H  −0.25367400000000     −2.01745700000000      9.26611900000000
H  −0.91576800000000     −2.25697600000000      7.85427200000000
H  −2.02057300000000      0.20285700000000      9.27256800000000
H  −2.27322700000000      0.85835100000000      7.85885200000000
H   2.19292900000000     −0.90084700000000      7.84034100000000
H   1.95583400000000     −0.23648100000000      9.25265100000000
Ni −0.01381700000000     −0.01357900000000     12.46686600000000
O   0.00874100000000      2.07368700000000     12.46373400000000
O  −0.03637600000000     −2.10084400000000     12.46999800000000
O  −2.10105600000000      0.00899600000000     12.47797600000000
O   2.07342100000000     −0.03615400000000     12.45575600000000
O  −0.00267400000000     −0.01056700000000     14.55422400000000
H  −0.20446700000000      2.00797800000000     13.43112100000000
H  −0.83640200000000      2.23577800000000     12.00402800000000
H   0.80170200000000     −2.26265900000000     11.99730900000000
H   0.18963000000000     −2.03785700000000     13.43414500000000
H  −2.00612200000000     −0.25664700000000     13.42965500000000
H  −2.25189000000000     −0.81135200000000     11.97094400000000
H   2.03307000000000      0.20290900000000     13.41620800000000
H   2.22615300000000      0.79372900000000     11.96595900000000
Ni  0.00846900000000     −0.00755500000000     16.64158200000000
O   0.03102800000000      2.07971000000000     16.63845000000000
O  −0.01409000000000     −2.09482100000000     16.64471400000000
O  −2.07877000000000      0.01502000000000     16.65269200000000
O   2.09570700000000     −0.03013000000000     16.63047200000000
O   0.01961200000000     −0.00454400000000     18.72894000000000
H   0.24618900000000      1.99654000000000     17.60551100000000
H   0.87753900000000      2.23823700000000     16.18025800000000
H  −0.20316400000000     −1.98539600000000     17.61641700000000
H  −0.87394900000000     −2.28246300000000     16.22544300000000
H  −2.24131200000000      0.73436000000000     17.29157100000000
H  −2.02179000000000      0.42803400000000     15.75703100000000
H   2.25299100000000     −0.89124500000000     16.20033700000000
H   2.01208600000000     −0.21473700000000     17.60427400000000
Ni  0.03075500000000     −0.00153200000000     20.81629800000000
O   0.05331400000000      2.08573400000000     20.81316700000000
O   0.00819600000000     −2.08879800000000     20.81943000000000
O  −2.05648300000000      0.02104300000000     20.82740799999999
O   2.11799300000000     −0.02410700000000     20.80518899999999
O   0.04189800000000      0.00148000000000     22.90365700000000
H  −0.13365900000000      1.97195000000000     21.78599100000000
H  −0.80056000000000      2.31563900000000     20.40355300000000
H   0.85286400000000     −2.26369000000000     20.36395100000000
H   0.22226100000000     −2.00639400000000     21.78700100000000
H  −2.27959700000000     −0.80626500000000     21.29138300000000
H  −1.94079800000000     −0.22395100000000     19.86854400000000
H   2.03665100000000      0.20156300000000     21.76970000000000
H   2.27999700000000      0.81572500000000     20.33572299999999
Ni  0.05304100000000      0.00449100000000     24.99101500000000
O   0.07560000000000      2.09175700000000     24.98788300000000
O   0.03048200000000     −2.08277400000000     24.99414600000000
O  −2.03419700000000      0.02706600000000     25.00212400000000
O   2.14027900000000     −0.01808400000000     24.97990500000000
O   0.06418400000000      0.00750300000000     27.07837299999999
H   0.33163700000000      2.00485700000000     25.94382400000000
H   0.90358100000000      2.23881800000000     24.49360600000000
H  −0.07789400000000     −1.95346600000000     25.97857899999999
H  −0.85649400000000     −2.29841500000000     24.65389900000000
H  −2.23130200000000      0.89705700000000     25.39449400000000
H  −1.90280800000000      0.20073400000000     24.02730700000000
H   2.21983900000000     −0.71421500000000     24.29704499999999
H   2.19233600000000     −0.46052300000000     25.85539600000000
Ni  0.07532700000000      0.01051500000000     29.16573100000000
O   0.09788600000000      2.09778000000000     29.16259900000000
O   0.05276800000000     −2.07675100000000     29.16886300000000
O  −2.01191100000000      0.03309000000000     29.17684100000000
O   2.16256600000000     −0.01206100000000     29.15462100000000
O   0.08647000000000      0.01352600000000     31.25308900000000
```

```
H  -0.07293000000000      2.05819600000000     30.13715000000000
H  -0.74399400000000      2.35381400000000     28.74525199999999
H  -0.80183200000000     -2.48598000000000     28.94747200000000
H   0.06413200000000     -1.93623400000000     30.15706000000000
H  -2.42682800000000     -0.76042400000000     29.55672300000000
H  -1.90522900000000     -0.12400800000000     28.19540399999999
H   2.41554200000000      0.89803800000000     29.39786599999999
H   1.98624600000000      0.02988300000000     28.17144599999999
Ni  0.09761300000000      0.01653800000000     33.34044700000000
O   0.12017200000000      2.10380300000000     33.33731500000000
O   0.07505400000000     -2.07072800000000     33.34357900000000
O  -1.98962500000000      0.03911300000000     33.35155700000000
O   2.18485200000000     -0.00603800000000     33.32933700000000
O   0.10875600000000      0.01955000000000     35.42780500000000
H   0.19155100000000      1.97000800000000     34.32004000000000
H  -0.80856900000000      2.36204400000000     33.19014400000000
H  -0.72673200000000     -2.53591000000000     33.63885400000000
H  -0.00348500000000     -1.93485800000000     32.35492300000000
H  -2.41188600000000     -0.78171500000000     33.65767100000000
H  -1.85160600000000     -0.04280100000000     32.36064700000000
H   2.42139600000000     -0.91894300000000     33.57767400000000
H   2.11417500000000     -0.01501600000000     32.33829400000000
Ni  0.11990000000000      0.02256300000000     37.51516300000000
O   0.14245800000000      2.10982900000000     37.51202900000000
O   0.09734100000000     -2.06470300000000     37.51829800000000
O  -1.96733900000000      0.04513800000000     37.52627700000000
O   2.20713800000000     -0.00001200000000     37.50405600000000
O   0.13104300000000      0.02557700000000     39.60252099999999
H   0.12540900000000      1.89931800000000     36.52886700000000
H  -0.62977000000000      2.67958800000000     37.68239200000000
H  -0.81795100000000     -2.36563700000000     37.67201299999999
H   0.16416100000000     -1.94158500000000     36.53238600000000
H  -2.47654000000000      0.80287400000000     37.86322900000000
H  -1.85644200000000      0.16481400000000     36.54112700000000
H   2.58636200000000      0.88609000000000     37.65710900000000
H   2.08783500000000     -0.04423100000000     36.51392000000000
H   0.61491500000000      0.77347200000000     39.99633099999998
H   0.59645200000000     -0.77319300000000     39.91338999999999
```

6. Molecular Geometry of $[Cu_2(\mu\text{-}F)(H_2O)_6]^{3+}$

```
Cu       0.000000000      0.000000000      1.499725020
Cu       0.000000000      0.000000000     -1.500274980
O        2.087386510      0.000000000     -1.500274983
O       -2.087386510      0.000000000     -1.500274983
O        0.000000000      0.000000000     -3.587665943
F        0.000000000      0.000000000     -0.000274980
H        2.683390184      0.000000000     -2.268411687
H        2.661817135      0.000000000     -0.709202196
H       -2.661817135      0.000000000     -0.709202196
H       -2.683390184      0.000000000     -2.268411687
H        0.000000000      0.785493669     -4.161056841
H        0.000000000     -0.785493669     -4.161056841
O        2.087386499      0.000000000      1.499725020
O       -2.087386499      0.000000000      1.499725020
O        0.000000000      0.000000000      3.587115984
H        2.698265094     -0.756296382      1.501134602
H        2.698265094      0.756296382      1.501134602
H       -2.698265094      0.756296382      1.501134602
H       -2.698265094     -0.756296382      1.501134602
H        0.778396866      0.000000000      4.169208711
H       -0.778396866      0.000000000      4.169208711
```

7. Molecular Geometry of $[Ni_2(\mu\text{-}O)_2(H_2O)_8]$

```
  O      2.111244    2.033518    1.927982
  O      1.923239   -0.200321    0.004890
  O      2.318936    0.101268    4.152153
  O     -0.165688    1.887211    0.052393
  O      0.230008    2.188800    4.199657
  O      0.042003   -0.045039    2.276565
  O      0.022315    4.121050    1.975486
  O      4.200171   -0.054014    1.880480
  Ni     0.032160    2.038005    2.126025
  Ni     2.121087   -0.049526    2.078522
  O     -2.046924    2.042493    2.324067
  O      2.130931   -2.132571    2.229061
  H      2.683947   -0.691659    4.586140
  H      1.337114   -0.077587    4.039435
  H      2.269476   -2.551487    3.096795
  H      1.147241   -1.978397    2.170979
  H      4.596879   -0.166647    0.998764
  H      4.048953    0.927636    1.970917
```

```
H      2.696908   -0.570953   -0.458143
H      2.118317    0.776942    0.126342
H      0.816788    2.063732    0.169471
H     -0.527966    2.682855   -0.378837
H      1.005772    3.965999    2.039254
H     -0.110557    4.539650    1.106744
H     -2.441684    2.150274    3.207469
H     -1.893974    1.061400    2.229567
H      0.038492    1.211116    4.071358
H     -0.548500    2.554550    4.658517
```

8. Molecular Geometry of the Co_3O_4 quantum cluster

```
Co  29.61956000000000     35.74774000000000     31.66229000000000
Co  32.68366000000000     36.76911000000000     32.68366000000000
Co  30.64093000000000     38.81184000000000     30.64093000000000
O   31.78047000000000     37.67230000000000     29.50139000000000
O   31.78047000000000     39.95137999999999     31.78047000000000
O   29.50139000000000     35.86592000000000     33.58685000000000
O   29.50139000000000     39.95137999999999     29.50139000000000
O   29.73774000000000     33.82319000000000     31.54412000000000
O   31.54412000000000     37.90865000000000     33.82320000000000
O   33.82320000000000     37.90865000000000     31.54412000000000
O   29.73774000000000     35.62957000000000     29.73774000000000
O   33.82320000000000     35.62957000000000     33.82320000000000
O   29.50139000000000     37.67230000000000     31.78047000000000
O   31.54412000000000     35.62957000000000     31.54412000000000
O   27.69501000000000     35.86592000000000     31.78047000000000
```
